# Constitutive EGFR Activation Induced by PTPRR Downregulation Confers Resistance to KRAS Inhibitors

**DOI:** 10.1158/2767-9764.CRC-25-0489

**Published:** 2026-04-02

**Authors:** Hiroaki Kanemura, Toshiyuki Takehara, Osamu Maenishi, Shuta Tomida, Natsumi Iwawaki, Kei Kunimasa, Tomohiro Nakayama, Satomi Watanabe, Shinichiro Suzuki, Kazuko Sakai, Koichi Azuma, Keita Kudo, Kazuto Nishio, Kazuhiko Nakagawa, Hidetoshi Hayashi, Takeshi Teramura, Kimio Yonesaka

**Affiliations:** 1 Department of Medical Oncology, Kindai University Faculty of Medicine, Sakai, Japan.; 2 Division of Cell Biology for Regenerative Medicine, Institute of Advanced Clinical Medicine, Kindai University Faculty of Medicine, Sakai, Japan.; 3 Department of Pathology, Kindai University Faculty of Medicine, Sakai, Japan.; 4 Center for Comprehensive Genomic Medicine, Okayama University Hospital, Okayama, Japan.; 5 Department of Thoracic Oncology, Osaka International Cancer Institute, Osaka, Japan.; 6 Department of Medical Oncology, Kishiwada City Hospital, Kishiwada, Japan.; 7 Department of Genome Biology, Kindai University Faculty of Medicine, Sakai, Japan.; 8 Center for Genomics, Life Science Research Institute, Kindai University, Sakai, Japan.; 9 Division of Respirology, Neurology, and Rheumatology, Department of Internal Medicine, Kurume University School of Medicine, Kurume, Japan.; 10 Department of Medical Oncology, National Hospital Organization Osaka Minami Medical Center, Kawachinagano, Japan.

## Abstract

KRAS^G12C^ inhibitors, such as sotorasib, show clinical efficacy for non–small cell lung cancer (NSCLC) positive for the G12C mutations of KRAS, but primary and acquired resistance to these drugs remains a clinical problem. In this study, we show that the development of resistance to sotorasib in KRAS^G12C^-positive NSCLC cells was mediated by constitutive activation of EGFR resulting from downregulation of the protein tyrosine phosphatase receptor type R (PTPRR). PTPRR has been identified as a physiologic regulator of ERK signaling in several cancer types. In our study, PTPRR was demonstrated to bind directly to EGFR, facilitating its dephosphorylation on tyrosine residues. Resumption of PTPRR expression in the resistant cells attenuated EGFR phosphorylation and restored sotorasib sensitivity. PTPRR downregulation was associated with gene promoter hypermethylation in the sotorasib-resistant cells and NSCLC tissue samples. Furthermore, low PTPRR expression in tumor specimens was associated with shorter progression-free and overall survival for patients with NSCLC treated with sotorasib. In contrast to sotorasib, high PTPRR expression was associated with a poor response to EGFR tyrosine kinase inhibitors in *EGFR*-mutated NSCLC, suggesting that PTPRR may broadly regulate EGFR dependence in NSCLC. Finally, dual blockade of KRAS^G12C^ and EGFR showed a substantial antitumor effect in a xenograft model of sotorasib-resistant NSCLC. This approach is therefore a rational therapeutic strategy for KRAS^G12C^-positive NSCLC, especially for tumors showing PTPRR downregulation.

**Significance:**

The current study shows that downregulation of PTPRR induces EGFR activation and resistance to KRAS^G12C^ inhibitors in NSCLC, suggesting dual KRAS-EGFR blockade as a rational therapy. PTPRR may help identify patient subgroups that would benefit from the addition of EGFR inhibitors to KRAS^G12C^-targeted therapies.

## Introduction

The RAS family of proteins comprises KRAS, HRAS, and NRAS and plays a key role in human cancer. Each RAS protein is a small GTPase that serves as a signaling switch and toggles between guanosine triphosphate (GTP)–bound (active) and guanosine diphosphate (GDP)–bound (inactive) states. In its GTP-bound state, RAS activates several downstream effector pathways—including the MAPK and PI3K pathways—and thereby promotes cell proliferation, migration, and survival (1–4). About 20% of all human malignancies harbor oncogenic mutations of the RAS family of genes, with *KRAS* alone accounting for 75% of these mutations (5). The most common *KRAS* mutations affect the glycine-12 residue of the encoded protein, which is replaced by cysteine (KRAS^G12C^) or aspartate (KRAS^G12D^). These mutations render the protein insensitive to the stimulatory effect of GTPase-activating protein on GTPase activity and therefore serve to maintain KRAS in the GTP-bound (active) form and to activate downstream signaling pathways that promote tumor cell growth (1–4).

The KRAS^G12C^ mutation has been detected in lung adenocarcinoma (14%), colorectal cancer (5%), and pancreatic tumors (1%; ref. 6). The first clinically available KRAS^G12C^ inhibitors, sotorasib and adagrasib, have been shown to have a 30% to 40% response rate and to give rise to a progression-free survival (PFS) of ∼6 months in individuals with KRAS^G12C^-positive non–small cell lung cancer (NSCLC; refs. 4, 7–9). These agents bind to an allosteric pocket near the mutant cysteine residue that has been designated the switch II pocket (2). These inhibitors require this cysteine residue for binding and suppress KRAS^G12C^ signaling and tumor cell growth by binding covalently and selectively to KRAS^G12C^-GDP (10). Although these compounds have improved treatment outcomes for individuals with KRAS^G12C^-positive NSCLC, the extent and duration of their effects are limited compared with those of EGFR and ALK inhibitors, which typically achieve response rates of ∼70% and ∼80% and a median PFS of 18.9 and 34.8 months, respectively (11–13). Furthermore, the response rates of these inhibitors in colorectal cancer and pancreatic ductal adenocarcinoma are only ∼10% to 30%, further indicative of the inadequacy of their therapeutic effects (14–16).

Concurrent mutations of *KEAP1*, *SMARCA4*, and *CDKN2A* have been identified as key drivers of early disease progression in patients treated with single-agent sotorasib or adagrasib (17). On-treatment feedback reactivation of the RAS-MAPK pathway, resulting from the new synthesis and GTP loading of KRAS^G12C^ or RTK-mediated activation of wild-type RAS, can compromise the clinical efficacy of KRAS^G12C^ inhibitors (18). In addition, secondary genetic alterations of RAS-MAPK pathway components, which often emerge in multiple genes simultaneously as a result of convergent tumor evolution, or transdifferentiation of adenocarcinoma into squamous histology can further drive disease progression and present additional challenges to the improvement of clinical outcomes (19–22). The high prevalence of primary and acquired resistance to KRAS^G12C^ inhibitors has led to the development of numerous combinations of these agents with other drugs that are being evaluated in clinical trials for their ability to overcome therapy resistance (23).

We here show that the expression of protein tyrosine phosphatase (PTP) receptor type R (PTPRR) was downregulated via methylation of its gene promoter in sotorasib-resistant cancer cells. This downregulation of PTPRR resulted in the activation of EGFR–MAPK (ERK) signaling and thereby conferred sotorasib resistance. We also show that dual blockade of KRAS^G12C^ and EGFR is a rational therapeutic strategy for KRAS^G12C^-positive cancer with reduced expression of PTPRR.

## Materials and Methods

### Cell culture and reagents

The KRAS^G12C^-mutant NSCLC cell lines NCI-H2122 (RRID: CVCL_1531), SW1573 (RRID: CVCL_1720), and NCI-H358 (RRID: CVCL_1559); the *EGFR*-mutated NSCLC cell line NCI-H1975 (RRID: CVCL_1511); and the KRAS^G12C^-mutant colorectal cancer cell line SW837 (RRID: CVCL_1729) were obtained from the ATCC. The authenticity of the cell lines was confirmed by short tandem repeat profiling in May 2021. All cell lines were regularly tested for *Mycoplasma* contamination using the MycoAlert Mycoplasma Detection Kit (Lonza), and the most recent test confirmed that the cell lines were *Mycoplasma*-free in November 2025. H2122, SW1573, H358, H1975, and SW837 cells were cultured under a humidified atmosphere of 5% CO_2_ at 37°C in RPMI 1640 (Sigma-Aldrich) supplemented with 10% FBS and 1% penicillin-streptomycin. Sotorasib, adagrasib and osimertinib were obtained from MedChemExpress, and cetuximab was from Bristol Myers Squibb.

### Cell viability assay

Cell viability was assessed with a CellTiter-Glo luminescence assay (cat. #G7571, Promega). Cells were seeded in 96-well round-bottom plates at a density of 2 × 10^3^/well, cultured for 24 hours, and then exposed to drugs for 72 hours before the assay. Luminescence was measured with a microplate reader (ARVO MX1420; PerkinElmer), and values were expressed as a percentage of that for untreated cells.

### Immunoblot analysis

Cells (1 × 10^6^ to 1 × 10^7^) were seeded in 90-mm PrimeSurface plates (Sumitomo Bakelite) and incubated overnight in RPMI 1640 supplemented with 2% FBS or treated with drugs as indicated before analysis. Immunoblot analysis was performed as described previously (24). The primary antibodies used are listed in Supplementary Table S1.

### Reverse transcription-quantitative PCR analysis

Total RNA was extracted from cells using the TRIzol reagent (Thermo Fisher Scientific) and was subjected to reverse transcription (RT) with a PrimeScript RT Master Mix Kit (Takara Bio). The resulting cDNA was subjected to real-time PCR analysis with Perfect Real-Time SYBR Green II (Takara Bio) and a Thermal Cycler Dice Real-Time System Single (Takara Bio). The protocol consisted of incubation at 95°C for 20 seconds, followed by 40 cycles of 95°C for 5 seconds and 60°C for 30 seconds. For quantitation of relative gene expression, the threshold cycle values were normalized by that for the housekeeping gene *GAPDH* and were then calibrated with the ΔΔCt method. Amplification of contaminating genomic DNA was limited by the use of primers designed to span at least one intron. Primer sequences are provided in Supplementary Table S2.

### Coimmunoprecipitation analysis

Cells were lysed in immunoprecipitation extraction buffer [25 mmol/L Tris-HCl (pH 7.5), 100 mmol/L NaCl, 0.5% Triton X-100] for 30 minutes on ice. The lysate was centrifuged to remove debris, and the resulting supernatant was incubated overnight at 4°C with primary antibodies conjugated to Dynabeads Protein G (Thermo Fisher Scientific) in Tris-buffered saline containing 0.02% Tween-20. The precipitated proteins were eluted from the beads with SDS sample buffer for immunoblot analysis. All antibodies used are listed in Supplementary Table S1.

### siRNA transfection

Cells were seeded at 50% to 60% confluency in 12-well plates and cultured for 24 hours before transient transfection for 72 hours with siRNAs mixed with the Lipofectamine RNAiMax reagent (Thermo Fisher Scientific). The cells were then transferred to six-well plates for immunoblot analysis or to 96-well plates for growth inhibition assays. A scrambled control siRNA and PTPRR-specific siRNAs (Supplementary Table S2) were obtained from Japan Bio Services.

### Establishment of PTPRR-overexpressing cell lines

The pPB[Exp]-Puro-CAG-3xFLAG/PTPRR[NM_001207016.1]-IRES-EGFP (VectorBuilder Inc.) vector or the pPB-CAG-EGFP-IRES-Puro vector was transfected into H2122 and H2122AR30 cells together with a plasmid encoding the piggyBac transposase using a NEPA21 electroporation system (Nepa Gene). These cells were cultured in DMEM supplemented with 10% FBS and puromycin (1 ng/mL) for 10 days.

### Colony formation assay

H2122 and H2122AR30 cells were seeded at a density of 1 × 10^3^ per well in six-well plates, cultured for 24 hours, and then treated with 1 μmol/L sotorasib, cetuximab (10 μg/mL), or both drugs for 28 days, with replacement of the medium every 3 days. The cells were then gently washed with PBS, fixed with acetic acid/methanol (1:7 vol/vol) for 5 minutes, washed again, and stained with 0.5% crystal violet for 2 hours at room temperature.

### Bisulfite sequencing

Genomic DNA was isolated from cells using a Quick-DNA Miniprep Plus Kit (Zymo Research) and from formalin-fixed, paraffin-embedded (FFPE) tumor specimens using an AllPrep DNA/RNA FFPE Kit (QIAGEN). The quality and quantity of the isolated DNA were determined using a NanoDrop 2000 device (Thermo Fisher Scientific) and a PicoGreen dsDNA Assay Kit (Thermo Fisher Scientific), respectively. The DNA was processed with an EpiTect Fast DNA Bisulfite Kit (QIAGEN), and the target *PTPRR* sequence in the samples obtained after bisulfite treatment was amplified by PCR using Takara EpiTaq HS (Takara Bio) and a Thermal Cycler Dice instrument (Takara Bio) with the primers (forward and reverse, respectively) 5′-TGA​ATT​AGG​TAG​TTT​TTA​AGA​GAG​A-3′ and 5′-TAA​AAT​CCT​TCT​TTA​CTC​CAA​ATT​TTA​T-3′. The incubation protocol comprised an initial incubation for 2 minutes at 95°C; 45 cycles of 20 seconds at 95°C, 30 seconds at 53°C, and 20 seconds at 72°C; and a final incubation for 5 minutes at 72°C. The PCR products were then used as templates. The sequencing primer hybridized close to the sequence of interest. Pyrosequencing was performed using PyroMark Gold Q24 reagents (QIAGEN) and a PyroMark Q24 instrument (QIAGEN). The resulting pyrogram was analyzed with pyrosequencing data analysis software (QIAGEN).

### Cell line–derived xenograft models

All animal experiments were approved by the Committee on Safety and Ethical Handling Regulations for Laboratory Animal Experiments of Kindai University and were performed in accordance with institutional guidelines. H2122 or H2122AR30 cells (5 × 10^6^) were injected subcutaneously into the right flank of 4- to 6-week-old male BALB/cAJcl-nu/nu mice (CLEA Japan). After the tumors had achieved a volume of 0.2 cm^3^, the mice were randomly assigned to treatment and control groups. The animals were treated orally with sotorasib (100 mg/kg per day) or intraperitoneally with cetuximab (40 mg/kg twice weekly). Tumor volume and body weight were measured twice weekly in a nonblinded manner. The mice were killed if the tumors became necrotic or grew to a volume of 3 cm^3^. Tumor volume was defined as follows: 0.5 × length × width^2^.

### Tumor specimens and clinical data

FFPE tissue specimens were obtained from individuals with NSCLC with *EGFR* activating mutations or KRAS^G12C^ who were treated at Kindai University Hospital, Kishiwada City Hospital, Izumi City General Hospital, or Osaka International Cancer Institute between 2018 and 2024. Most of the tumor specimens were collected from the lung during a bronchoscopic diagnostic biopsy or surgical resection. Clinical data were retrieved from the medical records of the patients. Overall survival (OS) was measured from treatment initiation to death from any cause, whereas PFS was measured from treatment initiation to clinical or radiographic progression or death.

### IHC staining

FFPE tissue sections (thickness of 4 μm) were depleted of paraffin, subjected to antigen retrieval by treatment with trypsin, and stained with antibodies to PTPRR (17937-1-AP; Proteintech) as previously described (25). Given that IHC has shown that PTPRR is present mostly in the cytoplasm and that its staining varies in intensity, we performed a quantitative analysis of PTPRR expression in tumor cells according to the methodology adopted for similar molecular staining patterns in previous studies (26). No staining in 100% of cells was defined as negative status. Weak, regardless of the percentage of positive cells, or strong cytoplasmic reactivity in <50% of cells was defined as low, and strong cytoplasmic reactivity in >50% of cells was defined as high. The expression of PTPRR in cancer cells was interpreted by a board-certified pathologist in a blinded manner.

### Statistical analysis

Comparisons of continuous variables between two groups were performed using the unpaired *t* test unless indicated otherwise, and those among more than two groups were performed with one-way ANOVA followed by Dunnett’s multiple comparison test. Differences in survival curves constructed by the Kaplan–Meier method were assessed with the log-rank test. Missing data were not imputed. All *P* values are based on a two-sided hypothesis. Statistical analysis was performed with Stata IC software version 14.2 (RRID: SCR_012763, StataCorp), and data were graphically depicted using Stata IC version 14.2 and GraphPad Prism 7.0 (RRID: SCR_002798, GraphPad Software). A *P* value of < 0.05 was considered statistically significant.

### Study approval

All animal experiments were performed in accordance with the Recommendations for the Handling of Laboratory Animals for Biomedical Research and in compliance with the Committee on Safety and Ethical Handling Regulations for Laboratory Animal Experiments of Kindai University. The analysis of human specimens was also conducted in accordance with the Declaration of Helsinki and was approved by the Institutional Review Boards of the participating facilities. Written informed consent was obtained from all participants after an opportunity to opt out of this study was provided.

## Results

### EGFR plays a pivotal role in resistance to sotorasib in KRAS^G12C^-positive NSCLC cells

**Figure 1.**
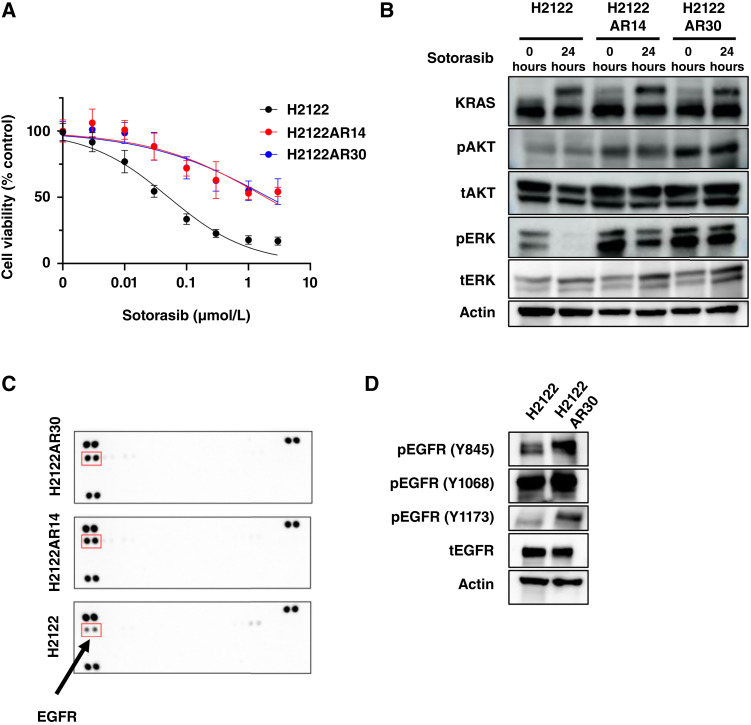
H2122AR14 and H2122AR30 cells are resistant to sotorasib *in vitro* and show EGFR activation. **A,** Cell viability assay for KRAS^G12C^-mutant parental H2122 cells and H2122AR14 and H2122AR30 sotorasib-resistant clones exposed to the indicated concentrations of sotorasib for 72 hours. Data are means ± SD (*n* = 3 independent experiments). **B,** Immunoblot analysis of KRAS, phosphorylated (p) and total (t) forms of AKT and ERK, and actin (loading control) in H2122, H2122AR14, and H2122AR30 cells treated with sotorasib (1 μmol/L) for 0 or 24 hours. **C,** Human phospho-RTK assay for H2122, H2122AR14, and H2122AR30 cells treated with 1 μmol/L sotorasib for 72 hours. **D,** Immunoblot analysis of Y845-, Y1068-, or Y1173-phosphorylated and total forms of EGFR in H2122 and H2122AR30 cells. Data in **B–D** are representative of at least three independent experiments.

To explore the mechanisms of sotorasib resistance, we generated resistant clones of KRAS^G12C^-mutant NSCLC (H2122) cells by exposing the parental cells to increasing concentrations of sotorasib over 10 months, as previously described (24). Whereas sotorasib exposure reduced the viability of parental H2122 cells in a concentration-dependent manner, the sotorasib-resistant clones—designated H2122AR14 and H2122AR30—remained viable in the presence of 1 μmol/L sotorasib (Fig. 1A). The IC_50_ of H2122AR14 (4.34 μmol/L) and H2122AR30 (4.79 μmol/L) cells was thus greater than that of the parental cells (0.05 μmol/L). Similar results were obtained with the KRAS^G12C^ inhibitor adagrasib, with the IC_50_ values of this drug being 0.03 μmol/L for parental H2122 cells and 9.35 μmol/L for H2122AR30 cells (Supplementary Fig. S1).

We next examined intracellular signaling in the KRAS^G12C^-mutant cells exposed to 1 μmol/L sotorasib (Fig. 1B). Immunoblot analysis revealed that sotorasib induced an upward shift of the KRAS band, reflecting the conversion of the GTP-bound (active) form to the GDP-bound (inactive) form, in both parental and sotorasib-resistant H2122 cells. These results thus suggested that sotorasib maintains KRAS^G12C^ in the inactive form in sotorasib-resistant H2122AR14 and H2122AR30 cells as well as in parental H2122 cells. Sotorasib also reduced the phosphorylation level of ERK but not that of AKT in the parental cells, indicating that sotorasib inhibited ERK signaling. However, sotorasib did not affect ERK phosphorylation in H2122AR14 or H2122AR30 cells.

Secondary alterations in on- or off-target genes in KRAS^G12C^-positive cancer cells have previously been shown to result in resistance to sotorasib through activation of ERK signaling (19, 21, 27). However, a next-generation sequencing panel detected no secondary gene alterations related to sotorasib resistance in sotorasib-resistant H2122AR14 or H2122AR30 cells (Supplementary Table S3), suggestive of a nongenetic mechanism of acquired resistance to sotorasib. We then assessed the activity of multiple RTKs that potentially activate ERK signaling using a human phospho-RTK array. Among the 49 RTKs examined, phosphorylation of EGFR was increased in H2122AR14 and H2122AR30 cells compared with parental H2122 cells (Fig. 1C). In addition, immunoblot analysis showed that phosphorylation of EGFR, in particular that at Y1173, was increased in H2122AR30 cells compared with the parental cells (Fig. 1D). These results suggested that constitutive activation of EGFR, potentially with contributions from other receptor tyrosine kinases or other signaling factors, may underlie ERK activation in the sotorasib-resistant H2122 cells.

### Expression of PTPRR is diminished in sotorasib-resistant cells

**Figure 2.**
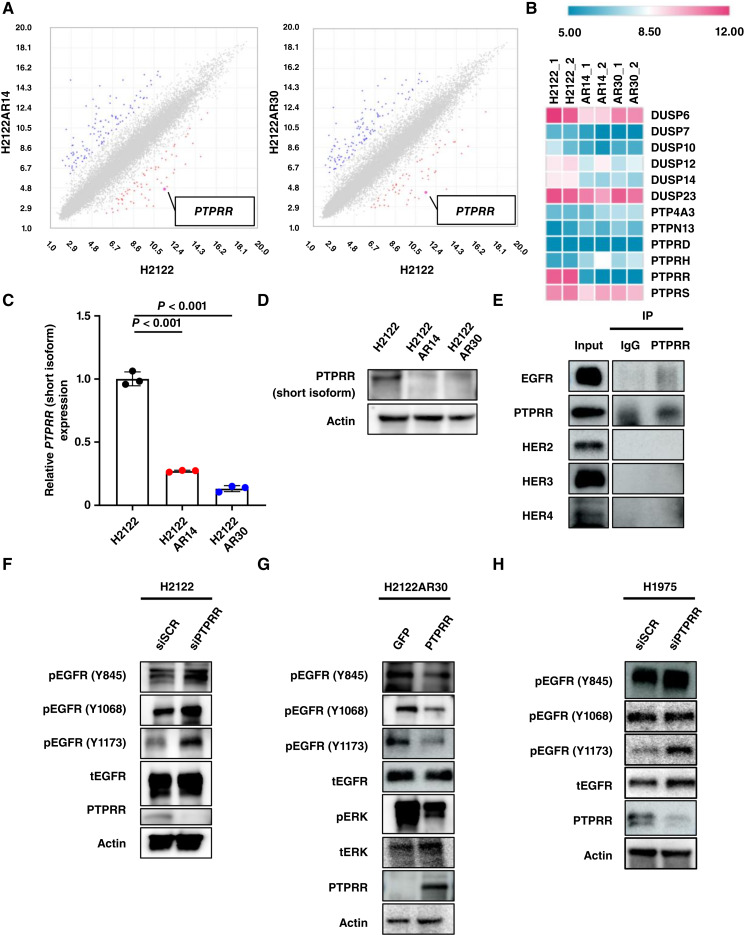
PTPRR is downregulated in sotorasib-resistant cells and mediates EGFR dephosphorylation. **A,** Scatter plots of gene expression in H2122 vs. H2122AR14 cells (left) or in H2122 vs. H2122AR30 cells (right) as revealed by microarray analysis. Each axis represents log_2_ intensity. Genes that are significantly up- or downregulated more than 10-fold in both sotorasib-resistant clones are shown in red and blue, respectively. **B,** Heatmap for the expression of phosphatase genes in two biological replicates of H2122, H2122AR14, and H2122AR30 cells determined as in **A**. **C,** RT-qPCR analysis of relative *PTPRR* mRNA abundance in H2122, H2122AR14, and H2122AR30 cells. Data are means ± SD (*n* = 3 independent experiments) and were analyzed by one-way ANOVA and Dunnett’s test. **D,** Immunoblot analysis of PTPRR in H2122, H2122AR14, and H2122AR30 cells. **E,** Lysates from H2122 cells transfected with PTPRR expression plasmids were subjected to immunoprecipitation (IP) with antibodies to PTPRR or with control IgG, and the resulting precipitates as well as the original cell lysates (Input) were subjected to immunoblot analysis with antibodies to either EGFR, PTPRR, HER2, HER3, or HER4. **F,** Immunoblot analysis of PTPRR as well as total and phosphorylated forms of EGFR in H2122 cells transfected with nonspecific control (siSCR) or PTPRR-specific (siPTPRR) siRNAs. **G,** Immunoblot analysis of PTPRR as well as total and phosphorylated forms of EGFR and ERK in H2122AR30 cells stably transfected with expression vectors for GFP (control) or PTPRR. **H,** Immunoblot analysis of PTPRR as well as total and phosphorylated forms of EGFR, AKT, and ERK in H1975 cells transfected with nonspecific control or PTPRR-specific siRNAs. Data in **D–H** are representative of at least three independent experiments.

To elucidate the molecular context of the increased phosphorylation of EGFR in sotorasib-resistant H2122 cells, we compared gene expression patterns between the resistant clones and the parental cells by microarray analysis. Assuming a shared resistance mechanism for H2122AR14 and H2122AR30 cells, we identified 126 genes with common expression changes in both cell lines (Supplementary Fig. S2). These up- or downregulated genes did not include those for EGFR ligands or other HER (human EGFR) family members. However, the gene for PTPRR was downregulated in the resistant clones relative to the parental cells (Fig. 2A and B; Supplementary Table S4). PTPRR exists as two major isoforms generated from distinct promoters; in this study, we primarily analyzed the short isoform, which is predominantly expressed in NSCLC. RT–quantitative PCR (RT-qPCR) analysis confirmed that the abundance of *PTPRR* mRNA was reduced in H2122AR14 and H2122AR30 cells compared with the parental cells (Fig. 2C), whereas immunoblot analysis showed that the amount of PTPRR protein was also downregulated in the resistant clones (Fig. 2D).

We next examined the potential functional relation between EGFR and PTPRR by coimmunoprecipitation analysis. Immunoprecipitation with antibodies to PTPRR revealed that PTPRR was associated with EGFR but not with other HER family members in H2122 cells (Fig. 2E). Given that PTPRR dephosphorylates tyrosine residues of substrate proteins, we investigated the effect of PTPRR knockdown by RNAi on the phosphorylation of EGFR in parental H2122 cells. Knockdown of PTPRR increased the phosphorylation of EGFR on tyrosine residues, most prominently at Y1173, whereas it did not affect total EGFR abundance (Fig. 2F). Conversely, overexpression of PTPRR reduced the phosphorylation of EGFR tyrosine residues in H2122AR30 cells (Fig. 2G). We also found that PTPRR knockdown increased tyrosine phosphorylation of EGFR in H1975 NSCLC cells, which harbor an activating mutation of EGFR (Fig. 2H). Collectively, these results suggested that PTPRR binds to EGFR and directly mediates its dephosphorylation at tyrosine residues.

### Downregulation of PTPRR confers resistance to sotorasib through activation of EGFR-ERK signaling

**Figure 3.**
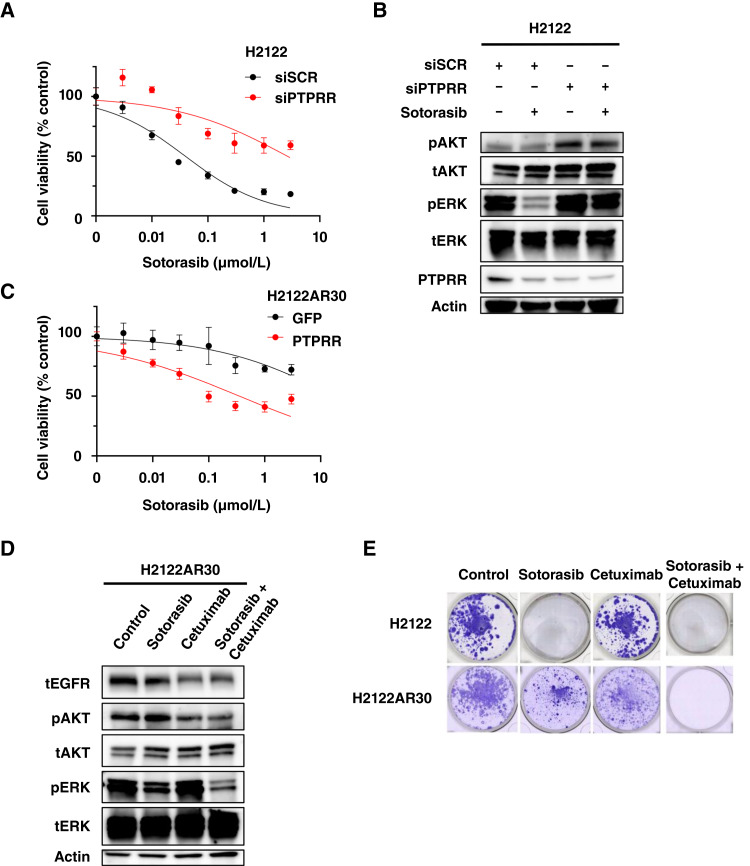
Combination treatment with EGFR and KRAS^G12C^ inhibitors overcomes sotorasib resistance due to PTPRR downregulation. **A,** Cell viability assay for H2122 cells transfected with nonspecific control (siSCR) or PTPRR-specific (siPTPRR) siRNAs and then exposed to the indicated concentrations of sotorasib for 72 hours. **B,** Immunoblot analysis of PTPRR as well as total and phosphorylated forms of AKT and ERK in H2122 cells transfected with siRNAs as in (**A**) and then incubated with or without sotorasib (1 μmol/L) for 4 hours. **C,** Cell viability assay for H2122AR30 cells stably transfected with control (GFP) or PTPRR expression plasmids and treated with the indicated concentrations of sotorasib for 72 hours. **D,** Immunoblot analysis of total or phosphorylated forms of EGFR, AKT, and ERK in H2122AR30 cells treated with sotorasib (1 μmol/L), cetuximab (10 μg/mL), or the combination of both drugs for 4 hours. **E,** Colony formation assay for H2122 and H2122AR30 cells incubated with or without sotorasib (1 μmol/L), cetuximab (10 μg/mL), or both drugs for 4 weeks. Surviving cells were stained with crystal violet. Data in **A** and **C** are means ± SD (*n* = 3 independent experiments), and those in **B**, **D**, and **E** are representative of at least three independent experiments.

To determine whether downregulation of PTPRR induces resistance to sotorasib, we examined sensitivity to the drug in PTPRR-depleted H2122 cells. PTPRR knockdown indeed reduced the sensitivity of these cells to sotorasib, with IC_50_ values of 4.2 and 0.041 μmol/L for the cells transfected with PTPRR and control siRNAs, respectively (Fig. 3A). Knockdown of PTPRR also increased the phosphorylation of AKT and ERK in H2122 cells (Fig. 3B). To assess whether this phenomenon was reproducible in additional KRAS^G12C^-mutant models, we performed similar experiments with another KRAS^G12C^-mutant NSCLC cell line (H358) as well as in the KRAS^G12C^-mutant colorectal cancer cell line SW837. In both H358 and SW837 cells, PTPRR knockdown similarly reduced sensitivity to sotorasib, indicating that PTPRR downregulation–mediated resistance is not restricted to the H2122 background (Supplementary Fig. S3A–S3D).

Sotorasib treatment reduced the phosphorylation of ERK in control cells but not in PTPRR knockdown cells (Fig. 3B). We then examined whether overexpression of PTPRR might restore the sensitivity of H2122AR30 cells to sotorasib. Overexpression of PTPRR indeed increased the sotorasib sensitivity of these cells compared with that apparent in control H2122AR30 cells expressing GFP, with IC_50_ values of 0.64 and 27.0 μmol/L, respectively (Fig. 3C).

We also investigated whether EGFR inhibition might affect intracellular signaling and restore sensitivity to sotorasib in H2122AR30 cells. Neither sotorasib nor the anti-EGFR antibody cetuximab alone had a substantial effect on the phosphorylation of ERK in H2122AR30 cells. Treatment with cetuximab alone resulted in partial inhibition of AKT phosphorylation, whereas sotorasib alone had no such effect. However, the combination of sotorasib and cetuximab attenuated the phosphorylation of both ERK and AKT (Fig. 3D). A cell viability assay also showed that cetuximab restored the anticancer effect of sotorasib in H2122AR30 cells (Supplementary Fig. S4A and S4B). We also performed a colony formation assay with H2122AR30 cells to evaluate the long-term efficacy of cetuximab. Colony formation by parental H2122 cells was suppressed by sotorasib alone, whereas that by H2122AR30 cells was not affected by sotorasib or cetuximab alone. Importantly, colony formation by H2122AR30 cells was abolished by exposure to the combination of sotorasib and cetuximab (Fig. 3E). Consistent with these findings, the combination of sotorasib and cetuximab also effectively overcame sotorasib resistance in PTPRR-downregulated SW837 cells, further supporting the role of EGFR signaling in mediating resistance downstream of PTPRR (Supplementary Fig. S5A and S5B). These results suggested that cetuximab was able to restore sensitivity to sotorasib by blocking EGFR-ERK signaling in PTPRR-deficient KRAS^G12C^-mutant NSCLC cells.

### PTPRR expression is regulated by promoter DNA methylation

**Figure 4.**
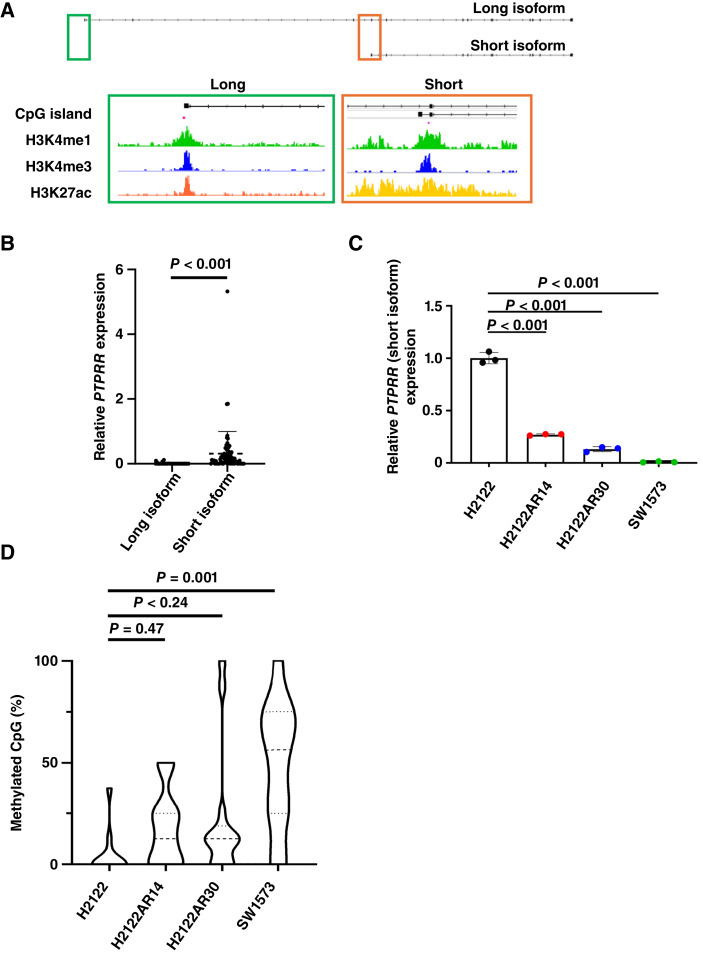
Expression of *PTPRR* is regulated by promoter DNA methylation in KRAS^G12C^-mutant NSCLC cell lines. **A,** The human *PTPRR* gene including the promoter regions for the long and short isoforms of the encoded protein is shown. The positions of CpG islands as well as the histone marks H3K4me1, H3K4me3, and H3K27ac are indicated. **B,** Abundance of transcripts for the long and short isoforms of PTPRR in tumor specimens from patients with NSCLC in the dbGaP database (*n* = 73). Data are presented as dot plots with means ± SD indicated by the dashed and solid lines for the short isoform, and they were analyzed with the Wilcoxon ranked-sum test. **C,** RT-qPCR analysis of mRNA abundance for the short form of PTPRR in the indicated KRAS^G12C^-mutant cell lines. Data are presented as dot plots, with means + SD being indicated by the dashed and solid lines (*n* = 3 independent experiments) and analyzed by one-way ANOVA and Dunnett’s test. **D,** Bisulfite genomic sequencing of the promoter region for the short variant of PTPRR in the indicated KRAS^G12C^-mutant cell lines. The percentage of methylated CpG sites among total CpG sites is shown. Data are presented as violin plots, with the median and upper quartile values indicated by the dashed and dotted lines and analyzed by one-way ANOVA and Dunnett’s test. Data are representative of three independent experiments.

PTPRR exists as two major variants in which expression is regulated by distinct promoters containing CpG islands (Fig. 4A; refs. 28–30). We found that the abundance of the mRNA for the short isoform of PTPRR was greater than that for the long form in NSCLC tumors of individuals in the dbGaP database (EAGLE study), with median values of 0.14 versus 0 (*n* = 73, *P* < 0.001); the short and long isoform mRNAs were detected in 76.7% (56 of 73) and 8.2% (6 of 73) of the tumor specimens (Fig. 4B). We then evaluated the relation between *PTPRR* expression and promoter methylation status in our H2122 cell lines as well as in SW1573, a KRAS^G12C^-mutant NSCLC cell line that is resistant to sotorasib. Transcripts encoding the long isoform of PTPRR were not detected in any of these cell lines by RT-qPCR analysis. The mRNA for the short form of PTPRR was also essentially undetectable in SW1573 cells (Fig. 4C). The extent of CpG methylation in the promoter region for the short variant of PTPRR, as determined by bisulfite sequencing, was greatly increased in H2122AR14, H2122AR30, and SW1573 cells compared with H2122 cells (Fig. 4D), and it was inversely related to the abundance of the short-form mRNA in these cells (Fig. 4C and D). These results suggested that promoter methylation is responsible for the downregulation of *PTPRR* expression in the sotorasib-resistant cells.

### EGFR blockade restores sensitivity to sotorasib in PTPRR-downregulated tumors *in vivo*

**Figure 5.**
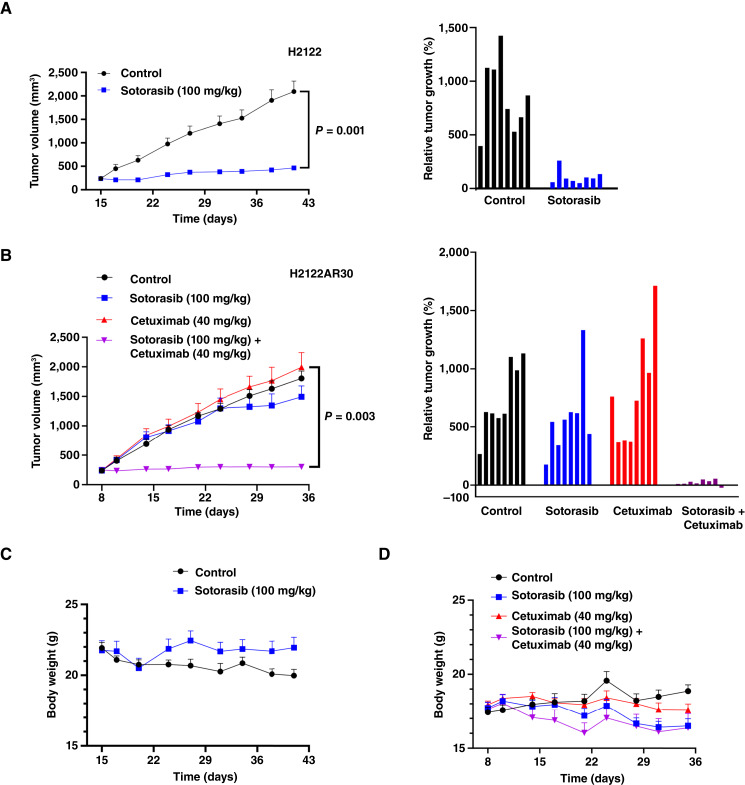
EGFR inhibition overcomes resistance to sotorasib in CDX tumors. Experiments were performed using H2122 (**A**) and PTPRR-downregulated H2122AR30 cells (**B**). **A,** Nude mice harboring H2122 xenografts were treated orally with sotorasib (100 mg/kg, daily) or vehicle (control). The time course of mean tumor volume (left) and relative tumor growth for individual tumors (*n* = 8) after treatment for 28 days (right) is shown. **B,** Nude mice harboring H2122AR30 xenografts were treated orally with sotorasib (100 mg/kg, daily) and i.p. with cetuximab (40 mg/kg, twice weekly) as indicated. The time course of mean tumor volume (left) and relative tumor growth for individual tumors (*n* = 8) after treatment for 28 days (right) is shown. **C** and **D,** Time course of mean body weight for tumor-bearing mice as in **A** and **B**, respectively. All time course data are means ± SEM. The *P* values for the indicated comparisons in **A** and **B** were determined using the unpaired *t* test.

To determine whether EGFR inhibition might restore sensitivity to sotorasib in PTPRR-downregulated cancer *in vivo*, we evaluated the anticancer effect of the combination of sotorasib and cetuximab in cell line–derived xenograft (CDX) models with H2122 and H2122AR30 cells. Consistent with our *in vitro* observations, sotorasib markedly attenuated the growth of H2122 cell xenografts in nude mice, with this effect persisting for at least 28 days (Fig. 5A). In contrast, neither sotorasib nor cetuximab alone suppressed the growth of H2122AR30 xenograft tumors (Fig. 5B). However, the administration of both drugs prevented the growth of these tumors for at least 4 weeks (Fig. 5B; Supplementary Fig. S6A and S6B). Toxicity, including body weight loss, was not greater for the combination treatment than for either type of monotherapy (Fig. 5C and D). These results thus suggested that the resistance to sotorasib conferred by downregulation of PTPRR in KRAS^G12C^-positive NSCLC can be overcome by combination therapy with sotorasib and cetuximab.

### Impact of PTPRR expression on clinical outcome in patients with NSCLC treated with sotorasib

**Figure 6.**
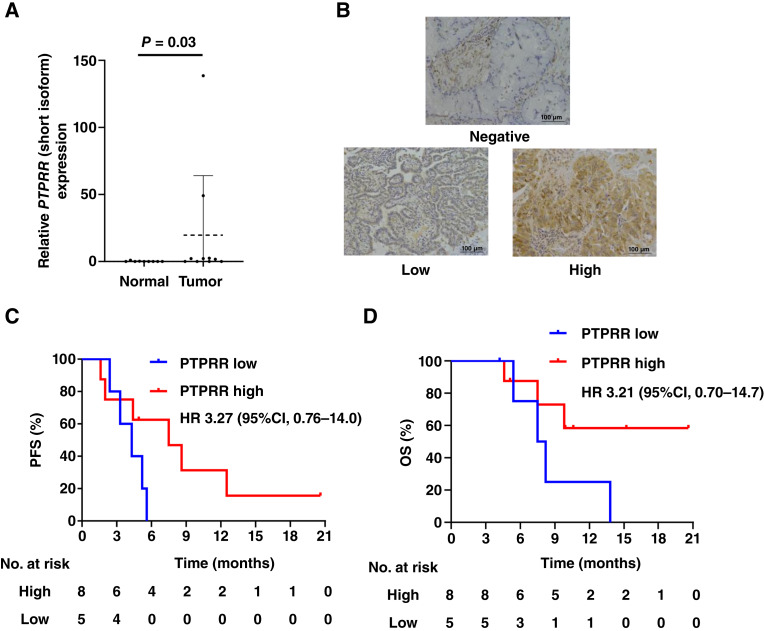
Clinical relevance of PTPRR expression to sotorasib treatment in individuals with NSCLC. **A,** RT-qPCR analysis of *PTPRR* mRNA (short form) abundance in normal lung tissue (*n* = 9) and NSCLC tumor tissue (*n* = 10). Data are presented as dot plots with means ± SD indicated by the dashed and solid lines, and they were analyzed with the Wilcoxon ranked-sum test. **B,** Representative IHC images for specimens of normal lung tissue (negative) and NSCLC tumor tissue (low and high) stained with antibodies to PTPRR. Scale bars, 100 μm. **C** and **D,** Kaplan–Meier analysis of PFS (**C**) and OS (**D**) according to low (*n* = 5) or high (*n* = 8) status for cytoplasmic PTPRR staining in tumor cells for patients with KRAS^G12C^-mutant NSCLC treated with sotorasib.

Given the role of PTPRR downregulation in resistance to KRAS^G12C^ inhibitors identified here in cell line models, we investigated the clinical relevance of PTPRR using human tumor specimens. RT-qPCR analysis revealed that the amount of *PTPRR* mRNA (short form) was significantly higher in NSCLC tumors (*n* = 10) than in normal lung tissue (*n* = 9) for the study cohort (median of 1.92 vs. 0, *P* = 0.03; Fig. 6A). Conversely, the DNA methylation level of the *PTPRR* promoter region (short form) was significantly lower in NSCLC tumor specimens than in normal lung tissue (median of 43 vs. 47, *P* = 0.04; Supplementary Fig. S7A). We also examined the methylation of the *PTPRR* promoter region (short form) for normal lung tissue (*n* = 68), peritumoral tissue (*n* = 65), and tumor tissue (*n* = 146) from patients with NSCLC in the dbGaP database. The methylation levels were found to be reduced in peritumoral (0.58 vs. 0.65, *P* = 0.01) and tumor (0.54 vs. 0.65, *P* < 0.001) tissue compared with normal tissue (Supplementary Fig. S7B). For a subset of cases with both RNA sequencing and methylation data available (*n* = 10), a trend toward a negative correlation between the abundance of *PTPRR* mRNA (short form) and the methylation of the *PTPRR* promoter region (short form) was apparent (Supplementary Fig. S7C).

We next examined the relationship between PTPRR expression and the clinical outcome of sotorasib treatment for 13 individuals with KRAS^G12C^-mutant NSCLC in the current study cohort. The expression of PTPRR in the cytoplasm of tumor cells was evaluated by IHC staining (Fig. 6B). Patient characteristics are summarized in Supplementary Table S5. Of the 13 pretreatment tumor specimens, 8 and 5 showed high and low PTPRR expression, respectively, with none of the specimens being negative for PTPRR expression. Patients with low PTPRR expression tended to have a shorter PFS on sotorasib treatment [median of 4.3 months (95% CI, 2.4 months to not reached) vs. 7.5 months (95% CI, 1.6–12.5 months), log-rank test *P* = 0.10; HR of 3.27, with a 95% CI of 0.76–14; Fig. 6C] and a shorter OS [median of 7.5 months (95% CI, 5.4 months to not reached) vs. not reached (95% CI, 4.6 months to not reached), log-rank test *P* = 0.13; HR of 3.21, with a 95% CI of 0.70–14.7; Fig. 6D] than those with high PTPRR expression. These observations thus suggested that a low level of PTPRR expression in tumor cells before treatment is associated with a poor clinical outcome of sotorasib treatment in patients with KRAS^G12C^-mutant NSCLC.

### Impact of PTPRR expression on clinical outcome in patients with NSCLC treated with EGFR tyrosine kinase inhibitors

Our *in vitro* data showed that PTPRR knockdown increased EGFR phosphorylation (Fig. 2H) as well as increased sensitivity to the EGFR tyrosine kinase inhibitor (TKI) osimertinib (Supplementary Fig. S8) in H1975 *EGFR* mutation–positive NSCLC cells, suggesting that PTPRR expression may also be associated with the therapeutic efficacy of EGFR-TKIs. We therefore evaluated the relationship between the abundance of *PTPRR* mRNA (short form) in pretreatment tumor specimens and the therapeutic efficacy of EGFR-TKIs in patients with *EGFR*-mutated NSCLC (*n* = 17). The characteristics of these patients are summarized in Supplementary Table S6. Individuals who acquired a secondary T790M mutation of *EGFR* were excluded because they tend to have a longer PFS than those without this mutation (31, 32). The amount of *PTPRR* mRNA in tumor specimens was determined by RNA-sequencing analysis (33). Patients with detectable *PTPRR* expression showed a significantly shorter PFS on EGFR-TKI treatment than those without such expression [median of 3 months (95% CI, 1.8 months to not reached) vs. 12.2 months (95% CI, 6.5–22.2 months), log-rank test *P* < 0.001; HR, not estimable; Supplementary Fig. S9]. Together, our preclinical and clinical observations suggested that *EGFR* activating mutation–positive NSCLC with downregulation of PTPRR may have a more favorable clinical outcome of EGFR-TKI treatment because such tumors are more dependent on EGFR signaling than those without PTPRR downregulation.

## Discussion

We have shown that downregulation of PTPRR conferred resistance to the KRAS^G12C^ inhibitor sotorasib in KRAS^G12C^-mutant NSCLC. Downregulation of PTPRR resulted in increased phosphorylation of EGFR on tyrosine residues, including Y1173 and consequent activation of the ERK signaling pathway. Expression of *PTPRR* seemed to be regulated by DNA methylation of the promoter region, with increased promoter methylation leading to sustained downregulation of PTPRR and constitutive activation of EGFR in sotorasib-resistant cells. Concomitant EGFR inhibition restored sensitivity to sotorasib in PTPRR-deficient cells, suggesting that the dual blockade of KRAS^G12C^ and EGFR is a rational therapeutic strategy for KRAS^G12C^-mutant tumors with reduced expression of PTPRR.

We and others previously showed that *MET* amplification gives rise to sotorasib resistance in NSCLC (20, 27). Such MET activation promotes ERK signaling independently of KRAS^G12C^ (20, 27), a situation similar to the present finding of EGFR-dependent resistance to sotorasib. However, although *MET* amplification does not seem to occur frequently in KRAS^G12C^-mutant cancers (20, 34), EGFR is frequently overexpressed in cancers, including NSCLC and colorectal cancer (35, 36). Of note, H2122 cells harbor not only KRAS^G12C^ but also *KEAP1* and *STK11* mutations, whereas SW1573 cells harbor KRAS^G12C^ together with the K111E mutation of *PIK3CA*. These comutations have been associated with a reduced sensitivity to KRAS^G12C^ inhibitors and may influence the dependence of these cells on EGFR (17, 37). In the present study, we found that EGFR activation was induced by downregulation of PTPRR in sotorasib-resistant cells. Consistent with this finding, EGFR-driven reactivation of MAPK/ERK signaling has previously been identified as a dominant mechanism of primary resistance to KRAS^G12C^ inhibition (18, 36). Furthermore, dual blockade of EGFR and KRAS^G12C^ signaling was previously found to be more effective than treatment with an EGFR inhibitor or KRAS^G12C^ inhibitor alone in some cancer cells harboring the KRAS^G12C^ mutation (15, 38, 39). Combined inhibition of EGFR and KRAS^G12C^ was also shown to have synergistic antitumor effects in NSCLC models (40, 41). Although various combination therapies, including a KRAS^G12C^ inhibitor, have been developed clinically, combinations with an EGFR inhibitor have shown efficacy in refractory cancers. A phase II trial (KROCUS) that evaluated the safety and efficacy of combination therapy with the KRAS^G12C^ inhibitor fulzerasib and cetuximab for treatment-naïve KRAS^G12C^-mutant NSCLC demonstrated a high response rate of 81.8% and a disease control rate of 100% (42). In addition, a phase Ib trial that assessed the combination of sotorasib and the anti-EGFR antibody panitumumab in individuals with previously treated KRAS^G12C^-mutant NSCLC reported a response rate of 47.5% (43). Sotorasib and adagrasib have also been approved by the FDA for combination therapy together with an EGFR inhibitor (panitumumab or cetuximab) for KRAS^G12C^-mutant colorectal cancer (15, 38, 39). Activation of EGFR by downregulation of PTPRR may underlie the efficacy of combined treatment with EGFR and KRAS inhibitors. Our preliminary examination of clinical specimens suggested that low PTPRR expression was associated with a poor clinical outcome in patients with NSCLC treated with the KRAS^G12C^ inhibitor sotorasib. The results obtained with our CDX models also indicated that the combination of an anti-EGFR antibody and KRAS^G12C^ inhibitor may be more effective than either monotherapy for PTPRR-deficient tumors.

Our study suggests that PTPRR plays a key role in the regulation of EGFR-ERK signaling in KRAS^G12C^-mutant cancer as well as in *EGFR* mutation–positive cancer. The extent of protein tyrosine phosphorylation is determined dynamically by the balance between the activities of protein tyrosine kinases and PTPs (44–46). The loss of PTPs, which can result from genetic alterations or epigenetic changes such as promoter DNA methylation (as in the case of PTPRR), has been shown to lead to the hyperactivation of signaling pathways (46). PTPRR has been identified as a physiologic regulator of ERK signaling in several cancer types (28, 47, 48). PTPRR interacts with multiple RTKs (49), but little has been known about its association with EGFR. Our results now suggest that PTPRR inhibits EGFR signaling by associating with EGFR and mediating its dephosphorylation and that *PTPRR* expression is regulated by promoter methylation. DNA methylation is an important mechanism of gene silencing and plays a pivotal role in cancer development. Inhibition of the MAPK pathway has been shown to disturb DNA methylation (50, 51). We speculate that inhibition of ERK signaling by sotorasib may therefore have disrupted DNA methylation and resulted in downregulation of PTPRR expression although such a mechanism was not directly demonstrated in the present study. Phosphorylation of EGFR on tyrosine residues, most prominently at Y1173, was increased in PTPRR-deficient cells; however, it remains unclear which tyrosine residues are directly targeted by PTPRR. Although phosphorylation at Y1173 was most pronounced, additional EGFR phosphorylation sites not assessed in the present study may play a role in the regulation of downstream ERK signaling, as previously described (52). Y1173 is the most COOH-terminal phosphorylation site of EGFR and activates downstream signaling through binding to the adapter protein SHC (53, 54). Knockout of PTPRH, another member of the PTP family, was recently shown to result in a selective increase in EGFR phosphorylation at Y1173 (bioRxiv 2024.06.13.598886), suggesting that individual PTP family members may differentially regulate phosphorylation at specific EGFR tyrosine residues. PTPRR may similarly influence EGFR phosphorylation in a site-specific manner. In addition to PTPRR, mitogen-induced gene 6 (MIG6, encoded by *ERRFI1*) has been identified as a negative regulator of EGFR, and its downregulation has been associated with resistance to ALK/ROS1 inhibitors mediated by EGFR-dependent signaling (55). Our microarray analysis did not reveal downregulation of *ERRFI1* expression in sotorasib-resistant cells. However, given that we did not examine MIG6 protein levels, we cannot conclude that the observed increase in EGFR phosphorylation is attributable solely to reduced PTPRR expression.

Our study has some limitations. First, the number of clinical specimens examined was small, and the results obtained with these specimens remain to be validated. Given the heterogeneity of tumors, larger-scale studies are warranted to clarify the relation between PTPRR expression and sotorasib efficacy in individuals with KRAS^G12C^-mutant NSCLC. The trends observed with clinical specimens from individuals with KRAS^G12C^-mutant and EGFR-mutant NSCLC did not achieve statistical significance and should, therefore, be interpreted as hypothesis-generating observations rather than as definitive conclusions. Second, the study did not evaluate changes in PTPRR expression before and after sotorasib treatment with paired pre- and posttreatment specimens, with future investigations needed to address this limitation. Third, mutations in the genes for other receptor-type PTPs—including PTPRT, PTPRD, and PTPRM—have been identified in various cancer types (56). Whether these genetic alterations influence EGFR dependency or interact with PTPRR downregulation warrants further investigation.

## Supplementary Material

Supplemental Methods Supplementary materials

Supplemental Figure 1 Cell viability of KRASG12C-mutant parental H2122 cells and the sotorasib-resistant clone H2122AR30 treated with adagrasib for 72 h.

Supplemental Figure 2 Venn diagram showing genes significantly upregulated or downregulated in H2122AR14 or H2122AR30 cells compared with parental H2122 cells based on microarray analysis.

Supplemental Figure 3 Effects of PTPRR knockdown on PTPRR mRNA expression and sotorasib sensitivity in H358 and SW837 cells.

Supplemental Figure 4 Effects of sotorasib and cetuximab, alone or in combination, on cell viability in the sotorasib-resistant H2122AR30 cells.

Supplemental Figure 5 Effects of sotorasib and cetuximab, alone or in combination, on cell viability in PTPRR-knockdown SW837 cells.

Supplemental Figure 6 Macroscopic tumor images and tumor weights from mice at the end of the treatment period in the experiment.

Supplemental Figure 7 Methylation status of the PTPRR promoter region and its correlation with PTPRR mRNA expression in normal lung and NSCLC tissues.

Supplemental Figure 8 Effects of PTPRR knockdown on sensitivity to osimertinib in H1975 cells.

Supplemental Figure 9 Kaplan–Meier analysis of progression-free survival according to PTPRR mRNA expression status in EGFR-mutant NSCLC patients treated with EGFR-TKIs.

Supplemental Table 1 Details of the antibodies used in this study

Supplemental Table 2 Sequences of RT-qPCR primers and siRNAs used in this study.

Supplemental Table 3 Summary of next-generation sequencing (NGS) results for parental H2122 cells and sotorasib-resistant H2122AR14 and H2122AR30 cells.

Supplemental Table 4 Genes commonly up- or downregulated in H2122AR14 and H2122AR30 cells relative to H2122 cells.

Supplemental Table 5 Baseline clinical characteristics of 13 NSCLC patients treated with sotorasib and classified according to PTPRR expression in pretreatment tumor specimens as determined by IHC.

Supplemental Table 6 Baseline clinical characteristics of 17 NSCLC patients treated with EGFR-TKIs and classified according to PTPRR mRNA abundance in pretreatment tumor specimens as determined by RNA-sequencing analysis.

## Data Availability

The data generated in this study are included in the main article and Supplementary information. Raw data are available on request without restriction from the corresponding author. Other data have been uploaded to NCBI Gene Expression Omnibus (https://www.ncbi.nlm.nih.gov/geo/) under data repository accession number GSE295133 and NCBI Sequence Read Archive (https://www.ncbi.nlm.nih.gov/sra) database under data repository BioProject accession number PRJNA1262108. Source data are provided with this article. Additional methods are included in Supplementary Methods.
