# Supplementary material for: Constitutive EGFR Activation Induced by PTPRR Downregulation Confers Resistance to KRAS Inhibitors
Source: Cancer Res Commun. 2026 Apr 2;6(4):728–41. doi: 10.1158/2767-9764.CRC-25-0489 (PMC13044349; doi:10.1158/2767-9764.CRC-25-0489)
Supplement: Supplemental Methods — Supplementary materials [file crc-25-0489_supplemental_methods_suppsm.doc]

**Supplemental Methods**

***Human phospho-RTK array analysis.*** The relative levels of phosphorylation of 49 kinases and 2 related total proteins were measured with a Human Phospho-Receptor Tyrosine Kinase Array Kit (R&D Systems, Minneapolis, MN).

***Microarray analysis.*** Total RNA was isolated from cells with the use of the TRI reagent (Molecular Research Center, Cincinnati, OH), treated with DNase, labeled with the use of a WTPLus Kit (Affymetrix, Santa Clara, CA), and probed with a Clariom S Human Array (Affymetrix). The raw CEL files were imported into the Transcriptome Analysis Console (TAC) software (version 4.0.2.15) (Thermo Fisher Scientific, Waltham, MA) for data processing and analysis. Gene-level expression values were calculated using the Signal Space Transformation Robust Multi-array Average (SST-RMA) algorithm for normalization and signal summarization.

***Next-generation sequencing (NGS) panel.*** An Oncomine Tumor Mutation Load Assay (Thermo Fisher Scientific) was adopted to test for single nucleotide variants, short insertions and deletions, and copy number variants of cancer-related genes. The assay fully covers the exons of 409 genes and was performed with DNA isolated from H2122, H2122AR14, and H2122AR30 cells.

***In silico* Chromatin immunoprecipitation (ChIP) assay.** *In silico* ChIP-sequence analysis was performed with GEO datasets for ChIP-seq databases for H3K4me1(SRX3236733), H3K4me3(SRX8848884), H3K27ac(SRX4143009 and DRX015259) of public database. Data analysis was performed using ChIP-Atlas (DBCLS, Tokyo, Japan and Kyusyu University, Fukuoka, Japan) and Integrative Genomics Viewer Ver. 2.3.90 (Broad Institute, Cambridge, MA, USA).

***Analysis of an NSCLC patient dataset******.*** Clinical, gene expression, and DNA methylation data for individuals with NSCLC corresponding to accession number phs001169.v2.p1(1,2) were downloaded with authorized access from the Database of Genotypes and Phenotypes (dbGaP). In the database, the PTPRR short and long isoforms are designated NM_001207016.1 and NM_002849.4, respectively. The mRNA abundance data (fastq data) related to the phs001169.v2.p1 study were selected via RunSelector and downloaded from SRA via SRA Toolkit. RNA-sequencing analysis was performed with CLC Genomics Workbench (Qiagen), and normalized transcripts per million (TPM) values were used for further analysis. The methylation region analyzed was cg25666210.

**References**

1. Hua X, Zhao W, Pesatori AC, Consonni D, Caporaso NE, Zhang T*, et al.* Genetic and epigenetic intratumor heterogeneity impacts prognosis of lung adenocarcinoma. *Nat Commun* 2020;**11**(1):2459 doi 10.1038/s41467-020-16295-5.

2. Zhao W, Zhu B, Hutchinson A, Pesatori AC, Consonni D, Caporaso NE*, et al.* Clinical Implications of Inter- and Intratumor Heterogeneity of Immune Cell Markers in Lung Cancer. *J Natl Cancer Inst* 2022;**114**(2):280-9 doi 10.1093/jnci/djab157.
