# Supplementary figures and images for: Constitutive EGFR Activation Induced by PTPRR Downregulation Confers Resistance to KRAS Inhibitors
Source: Cancer Res Commun. 2026 Apr 2;6(4):728–41. doi: 10.1158/2767-9764.CRC-25-0489 (PMC13044349; doi:10.1158/2767-9764.CRC-25-0489)

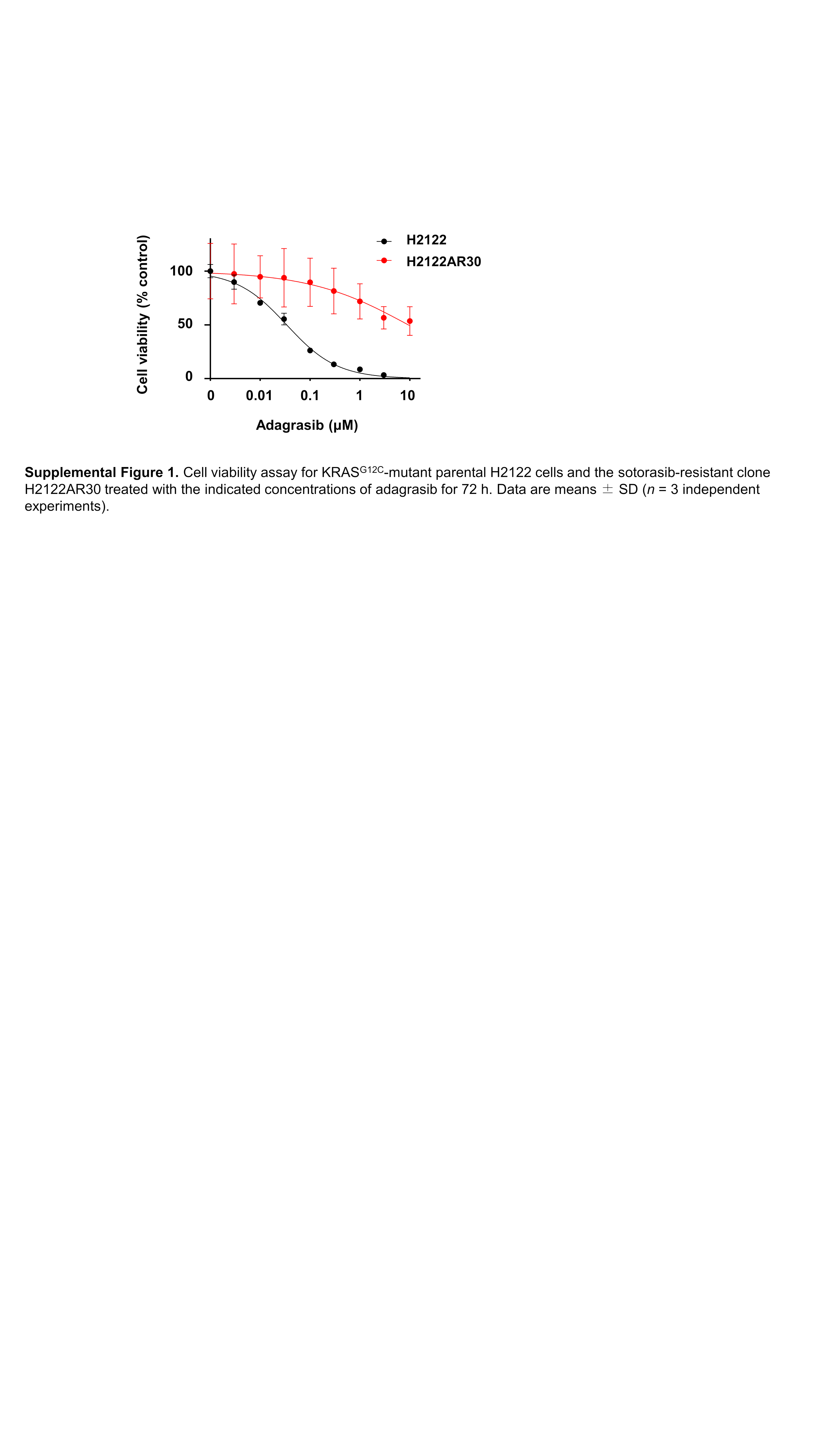

Supplement: Supplemental Figure 1 — Cell viability of KRASG12C-mutant parental H2122 cells and the sotorasib-resistant clone H2122AR30 treated with adagrasib for 72 h. [file crc-25-0489_supplemental_figure_1_suppsf1.png]

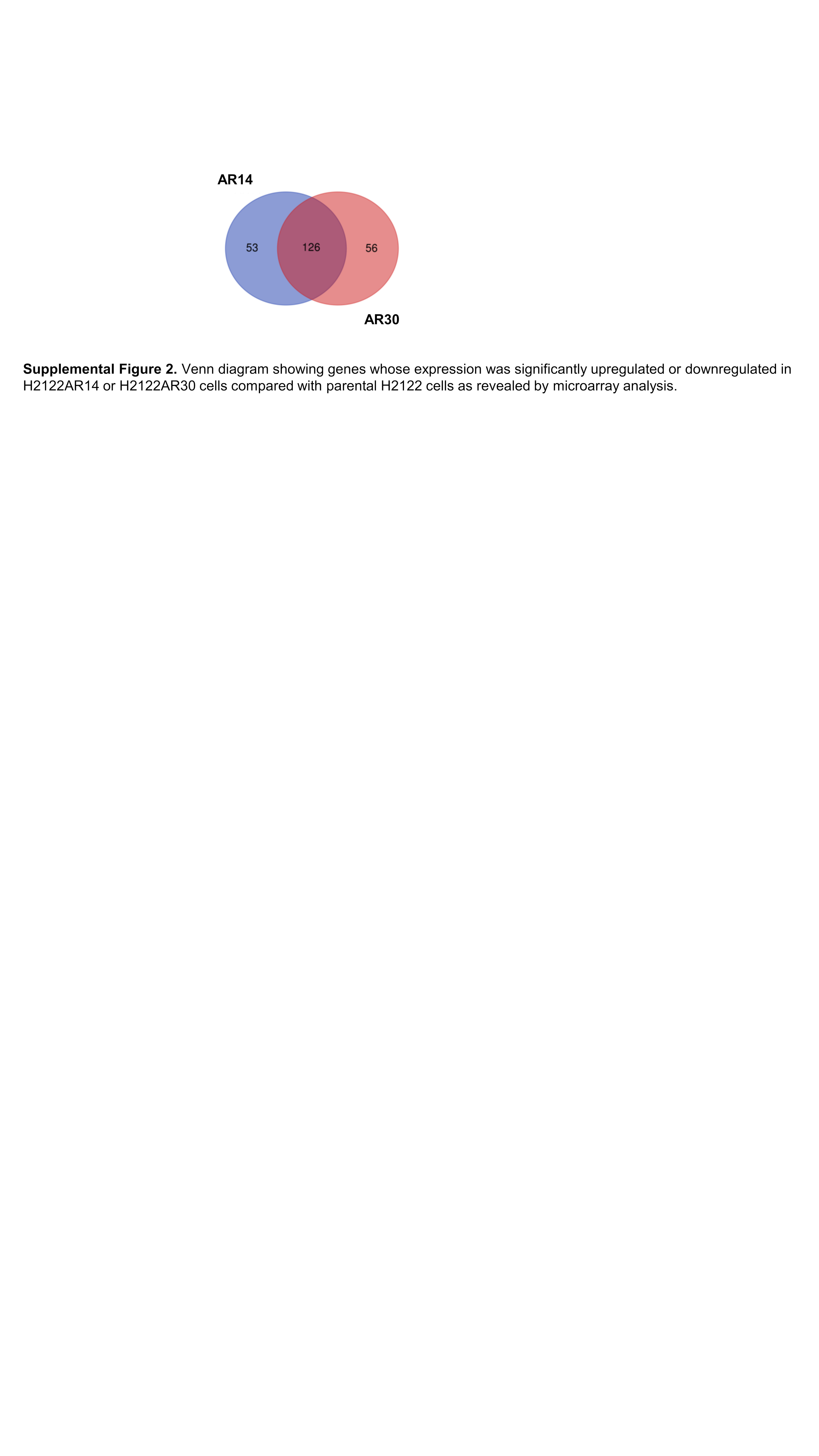

Supplement: Supplemental Figure 2 — Venn diagram showing genes significantly upregulated or downregulated in H2122AR14 or H2122AR30 cells compared with parental H2122 cells based on microarray analysis. [file crc-25-0489_supplemental_figure_2_suppsf2.png]

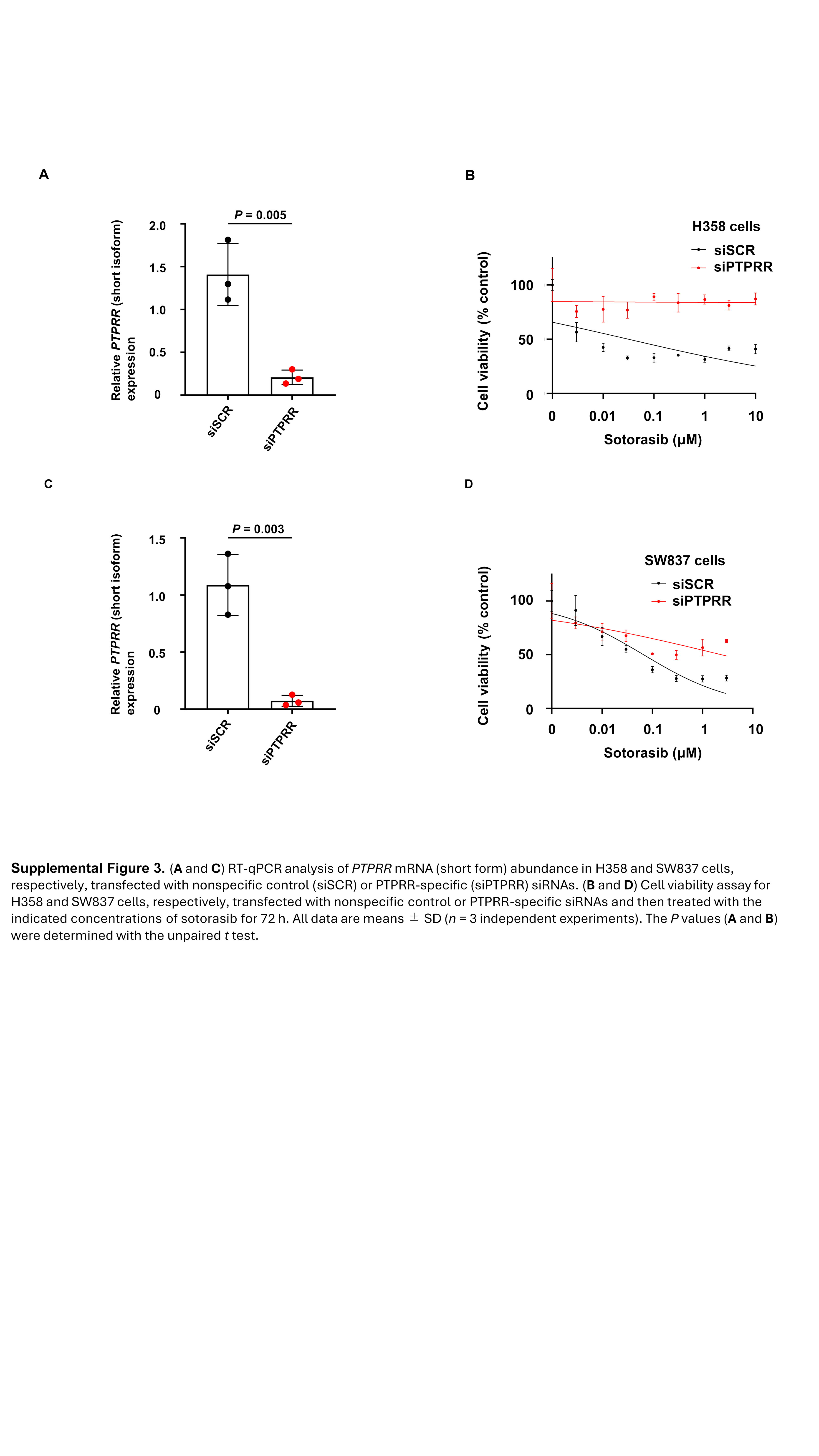

Supplement: Supplemental Figure 3 — Effects of PTPRR knockdown on PTPRR mRNA expression and sotorasib sensitivity in H358 and SW837 cells. [file crc-25-0489_supplemental_figure_3_suppsf3.png]

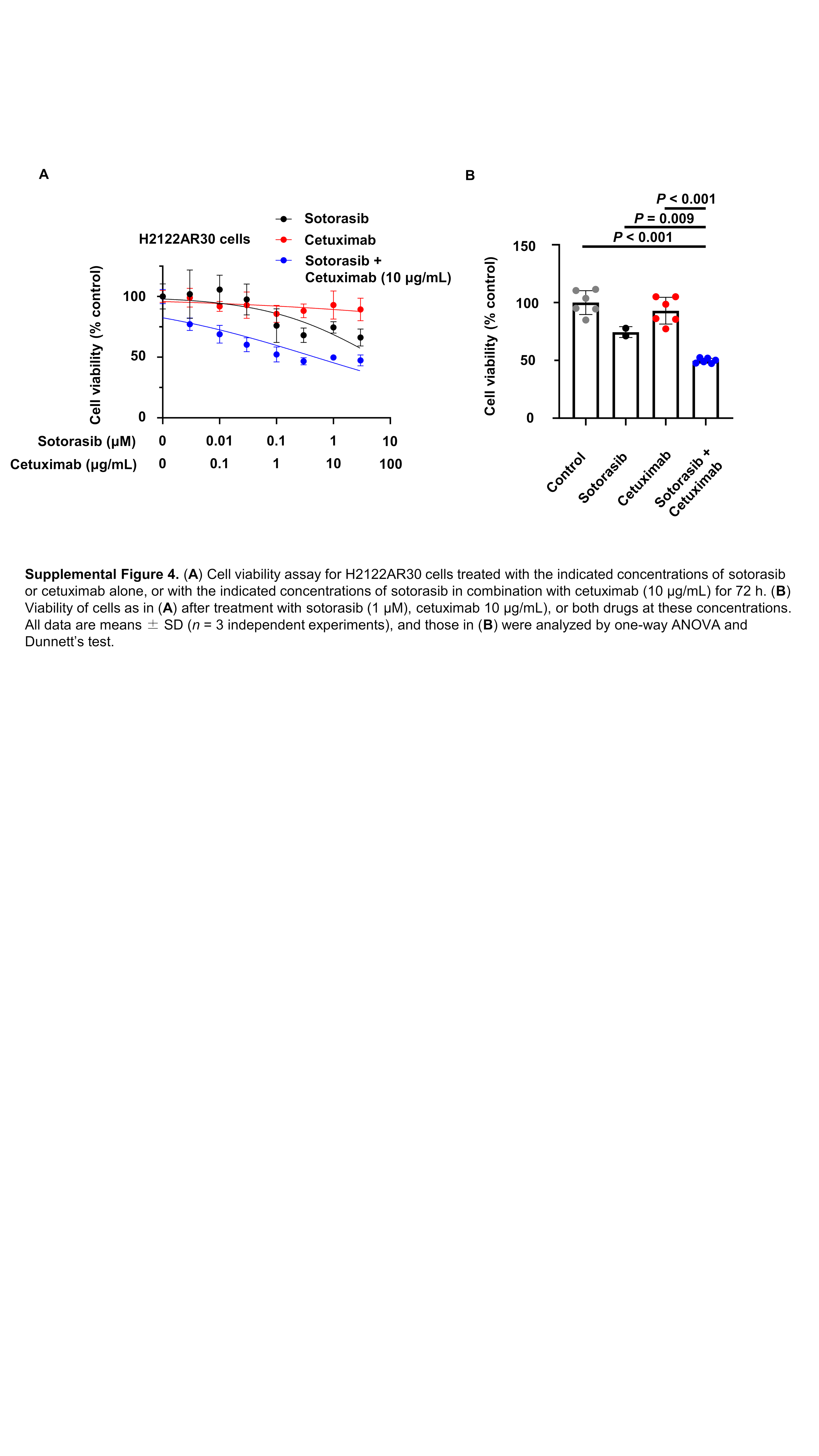

Supplement: Supplemental Figure 4 — Effects of sotorasib and cetuximab, alone or in combination, on cell viability in the sotorasib-resistant H2122AR30 cells. [file crc-25-0489_supplemental_figure_4_suppsf4.png]

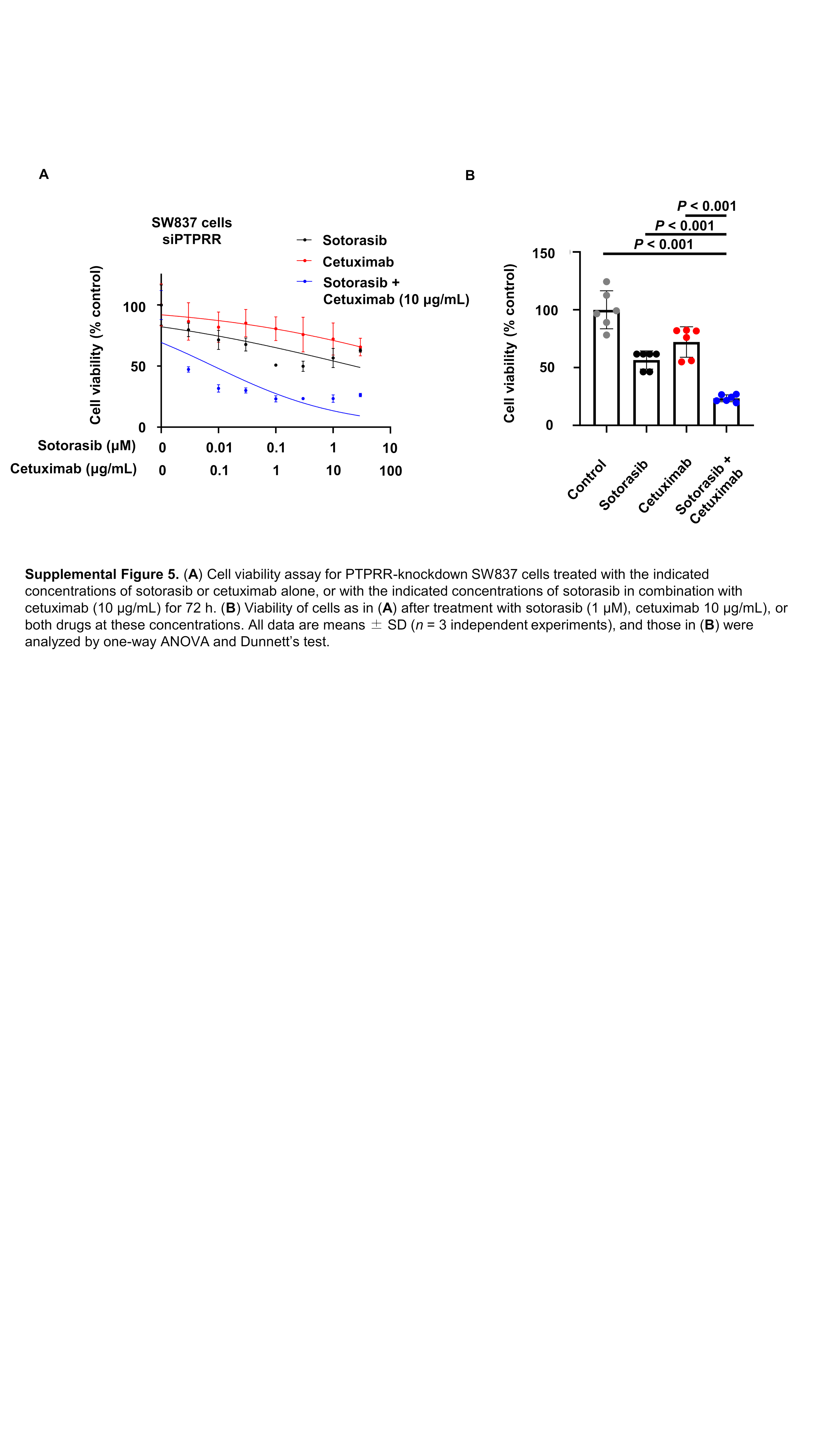

Supplement: Supplemental Figure 5 — Effects of sotorasib and cetuximab, alone or in combination, on cell viability in PTPRR-knockdown SW837 cells. [file crc-25-0489_supplemental_figure_5_suppsf5.png]

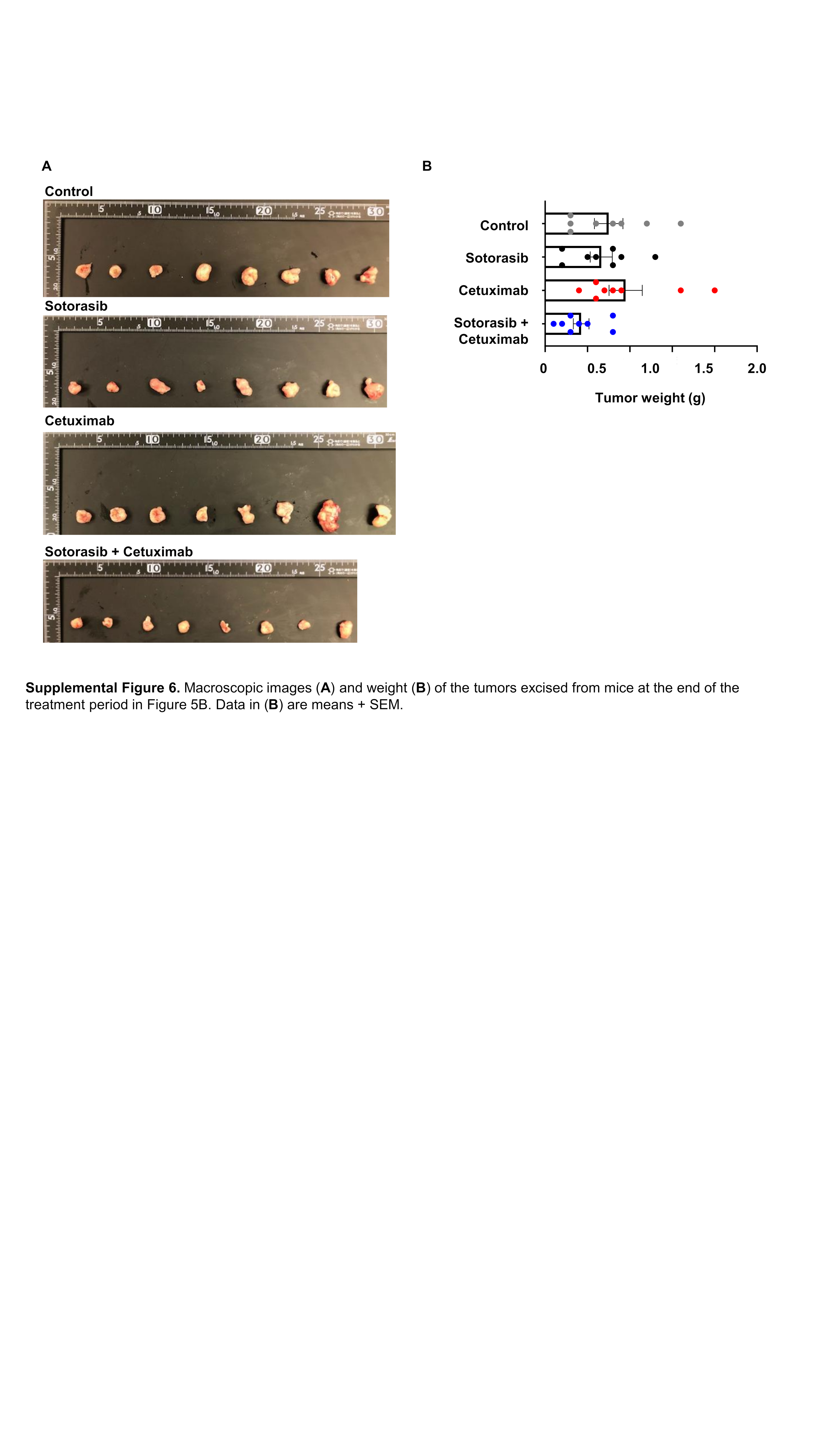

Supplement: Supplemental Figure 6 — Macroscopic tumor images and tumor weights from mice at the end of the treatment period in the experiment. [file crc-25-0489_supplemental_figure_6_suppsf6.png]

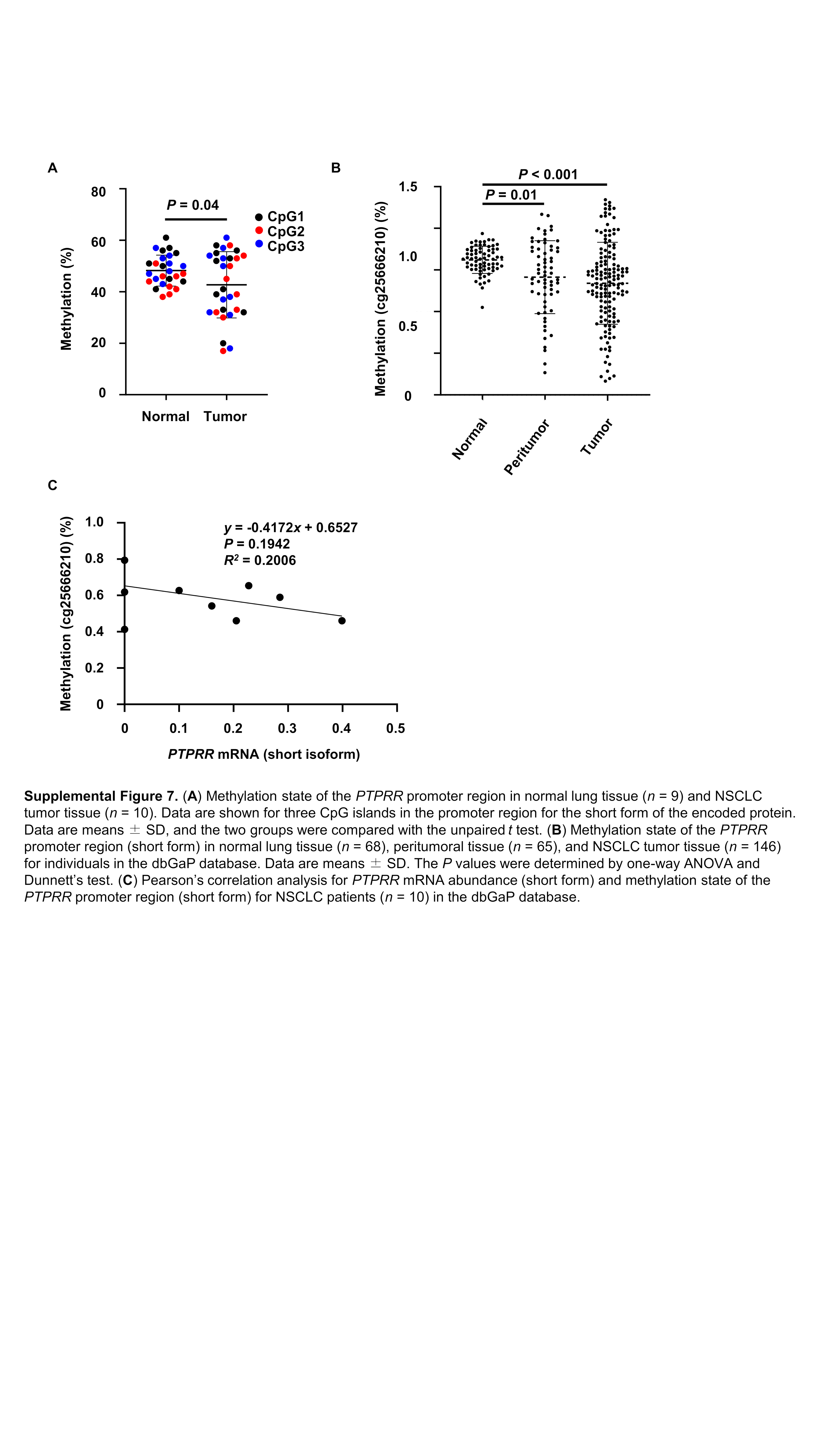

Supplement: Supplemental Figure 7 — Methylation status of the PTPRR promoter region and its correlation with PTPRR mRNA expression in normal lung and NSCLC tissues. [file crc-25-0489_supplemental_figure_7_suppsf7.png]

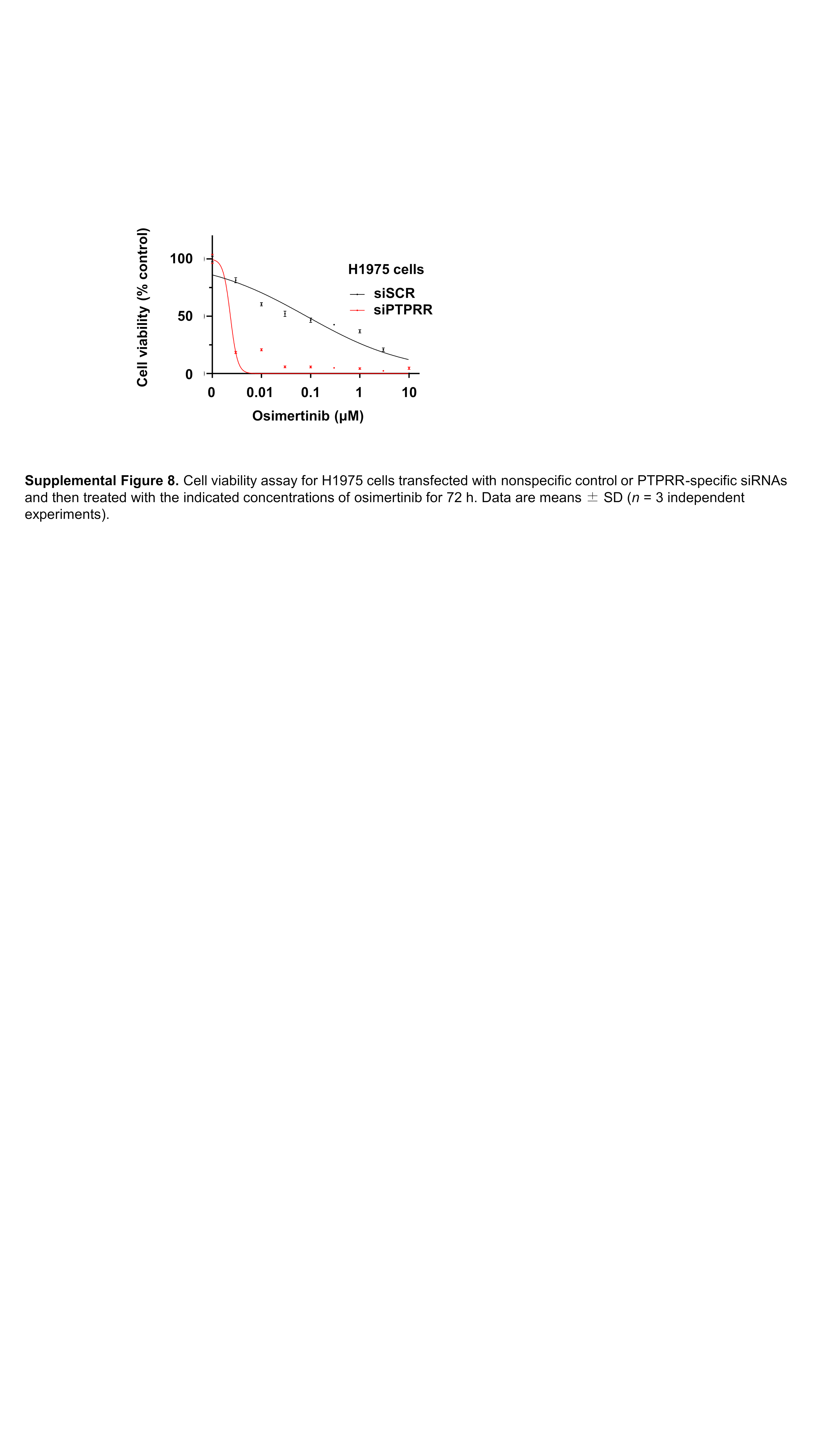

Supplement: Supplemental Figure 8 — Effects of PTPRR knockdown on sensitivity to osimertinib in H1975 cells. [file crc-25-0489_supplemental_figure_8_suppsf8.png]

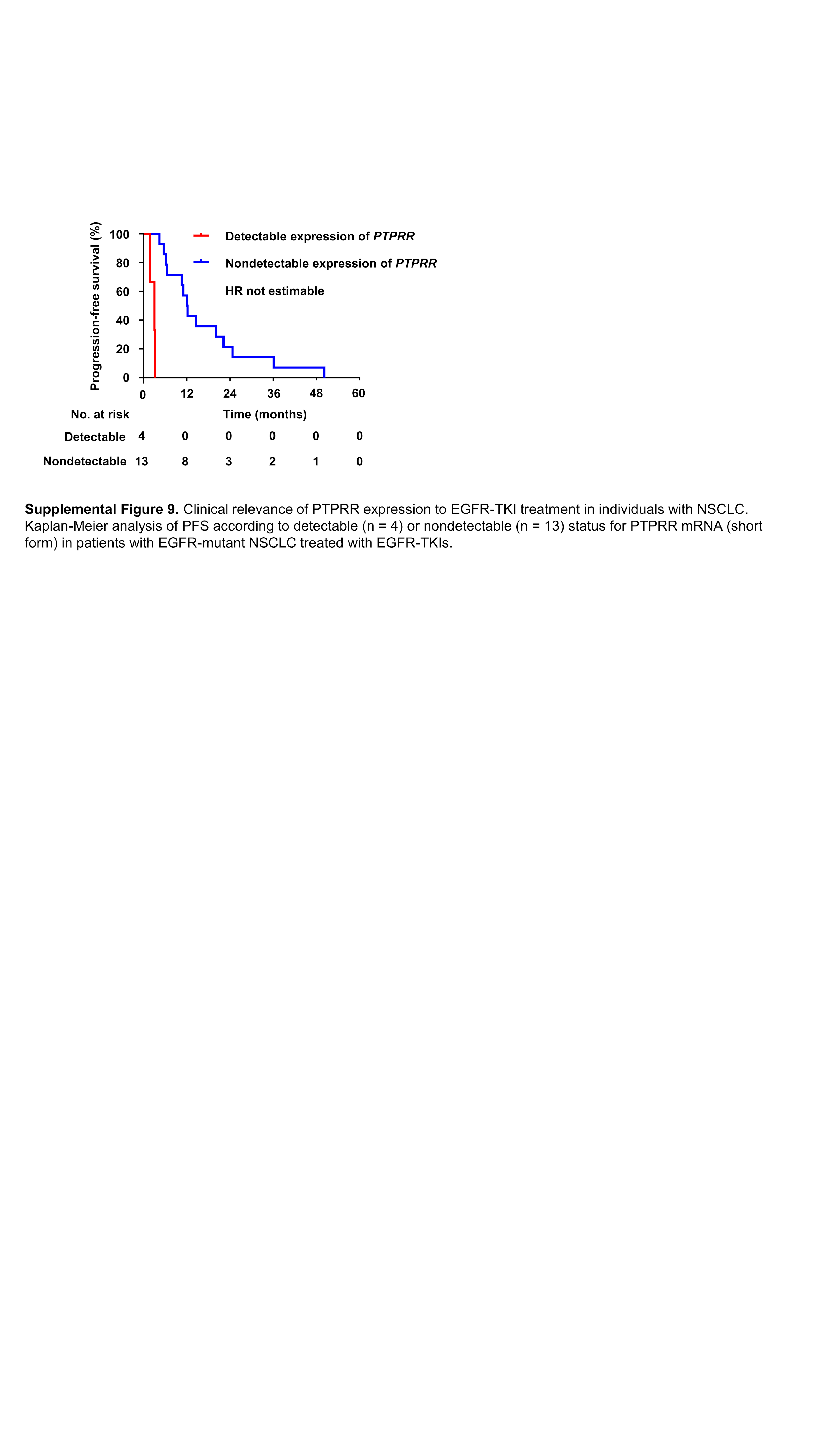

Supplement: Supplemental Figure 9 — Kaplan–Meier analysis of progression-free survival according to PTPRR mRNA expression status in EGFR-mutant NSCLC patients treated with EGFR-TKIs. [file crc-25-0489_supplemental_figure_9_suppsf9.png]
