# Supplementary material for: Constitutive EGFR Activation Induced by PTPRR Downregulation Confers Resistance to KRAS Inhibitors
Source: Cancer Res Commun. 2026 Apr 2;6(4):728–41. doi: 10.1158/2767-9764.CRC-25-0489 (PMC13044349; doi:10.1158/2767-9764.CRC-25-0489)
Supplement: Supplemental Table 1 — Details of the antibodies used in this study [file crc-25-0489_supplemental_table_1_suppst1.doc]

**Supplemental Table 1. Details of the antibodies used in this study**

| Target | Dilution | Company | Catalogue # |
| --- | --- | --- | --- |
| KRAS | 1:1000 | Sigma-Aldrich | SAB1404011 |
| PTPRR | 1:1000 | Proteintech | 17937-1-AP |
| pEGFR (Tyr^845^) | 1:1000 | Cell Signaling Technology | 2231 |
| pEGFR (Tyr^1068^) | 1:1000 | Cell Signaling Technology | 3777 |
| pEGFR (Tyr^1173^) | 1:1000 | Cell Signaling Technology | 4407 |
| tEGFR | 1:1000 | Cell Signaling Technology | 4267 |
| HER2 | 1:1000 | Cell Signaling Technology | 2165 |
| HER3 | 1:1000 | Santa Cruz Biotechnology | sc-285 |
| HER4 | 1:1000 | Santa Cruz Biotechnology | sc-283 |
| pAKT(Ser^473^) | 1:1000 | Cell Signaling Technology | 4060 |
| tAKT | 1:1000 | Cell Signaling Technology | 9272 |
| pERK1/2 (Thr^202^/Tyr^204^) | 1:1000 | Cell Signaling Technology | 4370 |
| tERK1/2 | 1:1000 | Cell Signaling Technology | 9102 |
| Actin | 1:1000 | Sigma-Aldrich | A2066 |
