# Supplementary material for: Constitutive EGFR Activation Induced by PTPRR Downregulation Confers Resistance to KRAS Inhibitors
Source: Cancer Res Commun. 2026 Apr 2;6(4):728–41. doi: 10.1158/2767-9764.CRC-25-0489 (PMC13044349; doi:10.1158/2767-9764.CRC-25-0489)
Supplement: Supplemental Table 2 — Sequences of RT-qPCR primers and siRNAs used in this study. [file crc-25-0489_supplemental_table_2_suppst2.doc]

**Supplemental Table 2. Sequences (5′-3′) of RT-qPCR primers and siRNAs used in this study**.

| RT-qPCR primers | Sequence | |
| --- | --- | --- |
| PTPRR long isoform | ATGCGGAGAGCAGTCTGCTTC  ACTCTTCTTCTGATTAATTGCC | |
| PTPRR short isoform | AGGCAAAGACAGTCCACAGC  TACGTTGGACCCTCTTCTCTC | |
| GAPDH | AAGGTGAAGGTCGGAGTC  TGTAGTTGAGGTCAATGAAGG | |
| siRNAs | Sequence |  |
| PTPRR-1 | Sense | GAA UGU AUG UAU GUA AUA UdTdT |
|  | Antisense | AUA UUA CAU ACA UAC AUU CdTdG |
| PTPRR-2 | Sense | GUA UCA AGA GUU UUA ACU AdTdT |
|  | Antisense | UAG UUA AAA CUC UUG AUA CdTdT |
