# Supplementary material for: Constitutive EGFR Activation Induced by PTPRR Downregulation Confers Resistance to KRAS Inhibitors
Source: Cancer Res Commun. 2026 Apr 2;6(4):728–41. doi: 10.1158/2767-9764.CRC-25-0489 (PMC13044349; doi:10.1158/2767-9764.CRC-25-0489)
Supplement: Supplemental Table 3 — Summary of next-generation sequencing (NGS) results for parental H2122 cells and sotorasib-resistant H2122AR14 and H2122AR30 cells. [file crc-25-0489_supplemental_table_3_suppst3.doc]

**Supplemental Table 3. Summary of next-generation sequencing (NGS) results for parental H2122 cells and sotorasib-resistant H2122AR14 and H2122AR30 cells.** The table lists gene alterations detected by the targeted NGS panel, highlighting differences between parental and resistant cell lines.

| Sample | Locus | Type | Genes | Coding change | Amino acid change | Allele frequency (%) | Cytoband | Copy number |
| --- | --- | --- | --- | --- | --- | --- | --- | --- |
| H2122AR14 | chr9:136918526 | MNV | BRD3 | c.73_74delGAinsAC | p.Glu25Thr | 13.0 | - | - |
| H2122AR14 | chr9:32631645 | INDEL | TAF1L | c.3932_3933insC | p.Val1312fs | 26.1 | - | - |
| H2122AR14 | chr11:4159572 | SNV | RRM1 | c.2338T>C | p.Ser780Pro | 4.5 | - | - |
| H2122AR14 | chr13:110435670 | SNV | IRS2 | c.2731C>G | p.Pro911Ala | 5.6 | - | - |
| H2122AR14 | chr14:105236757 | SNV | AKT1 | c.1364A>C | p.Asp455Ala | 3.2 | - | - |
| H2122AR14 | chr16:15820728 | INDEL | MYH11 | c.3855_3856insG | p.Asn1286fs | 3.7 | - | - |
| H2122AR14 | chr2:148602648 | CNV | ACVR2A, NFE2L2 | - | - | - | 2q22.3q31.2(148602648-178097329)x3 | 3 |
| H2122AR14 | chr2:16082166 | CNV | MYCN, MYCNOS | - | - | - | 2p24.3(16082166-16086219)x1 | 1 |
| H2122AR14 | chr22:36696832 | CNV | MYH9 | - | - | - | 22q12.3(36696832-36745288)x1 | 1 |
| H2122AR30 | chr4:1902847 | SNV | NSD2 | c.466G>T | p.Glu156Ter | 31.6 | - | - |
| H2122AR30 | chr6:117683803 | SNV | ROS1 | c.3344T>G | p.Leu1115Arg | 13.4 | - | - |
| H2122AR30 | chr7:2946367 | SNV | CARD11 | c.3370A>G | p.Met1124Val | 5.0 | - | - |
| H2122AR30 | chr9:120476201 | SNV | TLR4 | c.1795C>A | p.Gln599Lys | 45.8 | - | - |
| H2122AR30 | chrX:41075645 | SNV | USP9X | c.5825A>G | p.Gln1942Arg | 3.3 | - | - |
| H2122AR30 | chrX:70613199 | SNV | BCYRN1, TAF1 | c.3160T>C | p.Ser1054Pro | 3.7 | - | - |
| H2122AR30 | chr2:16082166 | CNV | MYCN, MYCNOS | - | - | - | 2p24.3(16082166-16086219)x1 | 1 |
| H2122AR30 | chr2:178095459 | CNV | NFE2L2 | - | - | - | 2q31.2(178095459-178129307)x3 | 3 |
| H2122AR30 | chr21:46306273 | CNV | ITGB2 | - | - | - | 21q22.3(46306273-46330714)x3 | 3 |

CNV, copy number variant; INDEL, insertion/deletion; MNV, multinucleotide variant; SNV, single-nucleotide variant.
