# Supplementary material for: Constitutive EGFR Activation Induced by PTPRR Downregulation Confers Resistance to KRAS Inhibitors
Source: Cancer Res Commun. 2026 Apr 2;6(4):728–41. doi: 10.1158/2767-9764.CRC-25-0489 (PMC13044349; doi:10.1158/2767-9764.CRC-25-0489)
Supplement: Supplemental Table 4 — Genes commonly up- or downregulated in H2122AR14 and H2122AR30 cells relative to H2122 cells. [file crc-25-0489_supplemental_table_4_suppst4.doc]

**Supplemental Table 4. Genes commonly up- or downregulated in H2122AR14 and H2122AR30 cells relative to H2122 cells.**

**Genes commonly upregulated in H2122AR14 and H2122AR30** cells

| Gene | H2122AR14 | | H2122AR30 | | Function |
| --- | --- | --- | --- | --- | --- |
|  | log_2_[fold change] | FDR | log_2_[fold change] | FDR |  |
| TM4SF20 | 12.2 | 0.0022 | 9.6 | 0.0055 | Cell proliferation, motility, and adhesion via interaction with integrins |
| TM4SF4 | 9.0 | 0.0007 | 6.4 | 0.0019 | Regulation of cell development, activation, growth, and motility |
| SLC4A4 | 8.0 | 0.0010 | 7.4 | 0.0013 | Regulation of bicarbonate secretion and absorption and intracellular pH |
| MUC3A | 7.4 | 0.0043 | 7.6 | 0.0035 | CLEC7A (Dectin-1) signaling and O-linked glycosylation of mucins |

**Genes commonly downregulated in H2122AR14 and H2122AR30** cells

| Gene | H2122AR14 | | H2122AR30 | | Function |
| --- | --- | --- | --- | --- | --- |
|  | log_2_[fold change] | FDR | log_2_[fold change] | FDR |  |
| SPANXB1 | -7.4 | 0.0001 | -6.7 | 0.0002 | Formation of mature spermatozoa |
| TFPI2 | -7.0 | 0.0003 | -6.8 | 0.0003 | Matrix metalloprotease and plasmin signaling |
| TNS4 | -6.8 | 0.0025 | -6.1 | 0.0036 | MET-promoted cell mobility and glucocorticoid receptor pathway |
| **PTPRR** | **-6.8** | **0.0008** | **-7.2** | **0.0006** | **Inhibition of ERK and PAK signaling** |
| DLX5 | -6.7 | 0.0010 | -6.3 | 0.0013 | Bone development and fracture healing |
| TRIM29 | -6.3 | 0.0001 | -8.8 | 0.0001 | DNA damage repair and interferon-γ signaling |
| ID3 | -6.1 | 0.0154 | -6.1 | 0.0149 | Nuclear events, NF-κB signaling, and Wnt/Hedgehog/Notch signaling |
| DKK1 | -5.9 | 0.0034 | -6.6 | 0.0020 | Pluripotent stem cell differentiation pathway |
