# Supplementary material for: Constitutive EGFR Activation Induced by PTPRR Downregulation Confers Resistance to KRAS Inhibitors
Source: Cancer Res Commun. 2026 Apr 2;6(4):728–41. doi: 10.1158/2767-9764.CRC-25-0489 (PMC13044349; doi:10.1158/2767-9764.CRC-25-0489)
Supplement: Supplemental Table 5 — Baseline clinical characteristics of 13 NSCLC patients treated with sotorasib and classified according to PTPRR expression in pretreatment tumor specimens as determined by IHC. [file crc-25-0489_supplemental_table_5_suppst5.doc]

**Supplemental Table 5. Baseline clinical characteristics of 13 NSCLC patients treated with s**otorasib and classified according to PTPRR expression in pretreatment tumor specimens as determined by IHC.

| Characteristic | Number of patients (%)^A^ | | |
| --- | --- | --- | --- |
|  | All patients  (*n* = 13) | Low group  (*n* = 5) | High group  (*n* = 8) |
| Median age (range), years | 72 (55–85) | 68 (57–85) | 73 (55–78) |
| Sex |  |  |  |
| Male | 12 (92.3) | 5 (100.0) | 7 (87.5) |
| Female | 1 (7.7) | 0 (0.0) | 1 (12.5) |
| ECOG performance status |  |  |  |
| 0 | 6 (46.2) | 0 (0.0) | 6 (75.0) |
| 1 | 7 (53.9) | 5 (100.0) | 2 (25.0) |
| Smoking history^B^ |  |  |  |
| Never | 0 (0.0) | 0 (0.0) | 0 (0.0) |
| Past or current | 13 (100.0) | 5 (100.0) | 8 (100.0) |
| Postoperative recurrence |  |  |  |
| Yes | 6 (46.2) | 3 (60.0) | 3 (37.5) |
| No | 7 (53.9) | 2 (40.0) | 5 (62.5) |

ECOG, Eastern Cooperative Oncology Group.

^A^Percentages may not add up to 100 because of rounding.

^B^Current smokers, individuals who had smoked a cigarette within the previous year; former smokers, those who had smoked ≥100 cigarettes but had quit >1 year before diagnosis; never-smokers, those who had smoked <100 cigarettes.
