# Supplementary material for: Constitutive EGFR Activation Induced by PTPRR Downregulation Confers Resistance to KRAS Inhibitors
Source: Cancer Res Commun. 2026 Apr 2;6(4):728–41. doi: 10.1158/2767-9764.CRC-25-0489 (PMC13044349; doi:10.1158/2767-9764.CRC-25-0489)
Supplement: Supplemental Table 6 — Baseline clinical characteristics of 17 NSCLC patients treated with EGFR-TKIs and classified according to PTPRR mRNA abundance in pretreatment tumor specimens as determined by RNA-sequencing analysis. [file crc-25-0489_supplemental_table_6_suppst6.doc]

**Supplemental Table 6. Baseline clinical characteristics of 17 NSCLC patients treated with EGFR-TKIs and classified according to *PTPRR* mRNA abundance in pretreatment tumor specimens as determined by RNA-sequencing analysis.**

| Characteristic | Number of patients (%)^A^ | | |
| --- | --- | --- | --- |
|  | All patients  (*n* = 17) | Nondetectable group  (*n* = 13) | Detectable group  (*n* = 4) |
| Median age (range), years | 72 (58–81) | 72 (58–77) | 74 (70–81) |
| Sex |  |  |  |
| Male | 6 (35.3) | 5 (38.5) | 1 (25.0) |
| Female | 11 (64.7) | 8 (61.5) | 3 (75.0) |
| ECOG performance status |  |  |  |
| 0 | 3 (17.6) | 2 (15.4) | 1 (25.0) |
| 1 | 13 (76.5) | 11 (84.6) | 2 (50.0) |
| 2 | 1 (5.9) | 0 (0.0) | 1 (25.0) |
| Smoking history^B^ |  |  |  |
| Never | 11 (64.7) | 8 (61.5) | 3 (75.0) |
| Past or current | 6 (35.3) | 5 (38.5) | 1 (25.0) |
| *EGFR* mutation |  |  |  |
| Exon 19 deletion | 5 (29.4) | 4 (30.8) | 1 (25.0) |
| L858R | 10 (58.8) | 8 (61.5) | 2 (50.0) |
| Other | 2 (11.8) | 1 (7.7) | 1 (25.0) |
| EGFR TKI for first-line treatment |  |  |  |
| First generation | 11 (64.7) | 9 (69.2) | 2 (50.0) |
| Second generation | 5 (29.4) | 4 (30.8) | 1 (25.0) |
| Third generation | 1 (5.9) | 0 (0.0) | 1 (25.0) |
| *TP53* status |  |  |  |
| WT | 7 (41.2) | 6 (46.2) | 1 (25.0) |
| Mutant | 8 (47.1) | 6 (46.2) | 2 (50.0) |
| Assay failure | 2 (11.8) | 1 (7.7) | 1 (25.0) |
| Postoperative recurrence |  |  |  |
| Yes | 2 (11.8) | 2 (15.4) | 0 (0.0) |
| No | 15 (88.3) | 11 (84.6) | 4 (100.0) |

ECOG, Eastern Cooperative Oncology Group.

^A^Percentages may not add up to 100 because of rounding.

^B^Current smokers, individuals who had smoked a cigarette within the previous year; former smokers, those who had smoked ≥100 cigarettes but had quit >1 year before diagnosis; never-smokers, those who had smoked <100 cigarettes.
